# Microbial biotransformation of DON: molecular basis for reduced toxicity

**DOI:** 10.1038/srep29105

**Published:** 2016-07-06

**Authors:** Alix Pierron, Sabria Mimoun, Leticia S. Murate, Nicolas Loiseau, Yannick Lippi, Ana-Paula F. L. Bracarense, Gerd Schatzmayr, Jian Wei He, Ting Zhou, Wulf-Dieter Moll, Isabelle P. Oswald

**Affiliations:** 1 Toxalim, Research Center in Food Toxicology, Université de Toulouse, INRA, UMR 1331, ENVT, INP-Purpan Toulouse, France; 2 Université de Toulouse, INP, UMR 1331, Toxalim, Toulouse, France; 3 BIOMIN Research Center Technopark 1, 3430 Tulln, Austria; 4 Universidade Estadual de Londrina, Lab. Patologia Animal, CP 6001, Londrina, Paraná, Brazil; 5 Guelph Food Research Center Agriculture & Agri-Food Canada, Guelph, Ontario N1G 5C, Canada

## Abstract

Bacteria are able to de-epoxidize or epimerize deoxynivalenol (DON), a mycotoxin, to deepoxy-deoxynivalenol (deepoxy-DON or DOM-1) or 3-epi-deoxynivalenol (3-epi-DON), respectively. Using different approaches, the intestinal toxicity of 3 molecules was compared and the molecular basis for the reduced toxicity investigated. In human intestinal epithelial cells, deepoxy-DON and 3-epi-DON were not cytotoxic, did not change the oxygen consumption or impair the barrier function. In intestinal explants, exposure for 4 hours to 10 μM DON induced intestinal lesions not seen in explants treated with deepoxy-DON and 3-epi-DON. A pan-genomic transcriptomic analysis was performed on intestinal explants. 747 probes, representing 323 genes, were differentially expressed, between DON-treated and control explants. By contrast, no differentially expressed genes were observed between control, deepoxy-DON and 3-epi-DON treated explants. Both DON and its biotransformation products were able to fit into the pockets of the A-site of the ribosome peptidyl transferase center. DON forms three hydrogen bonds with the A site and activates MAPKinases (mitogen-activated protein kinases). By contrast deepoxy-DON and 3-epi-DON only form two hydrogen bonds and do not activate MAPKinases. Our data demonstrate that bacterial de-epoxidation or epimerization of DON altered their interaction with the ribosome, leading to an absence of MAPKinase activation and a reduced toxicity.

Mycotoxins are toxic secondary metabolites produced by various molds, such as *Aspergillus, Penicillium* and *Fusarium* which may contaminate food and feed at all stages of the food/feed chain^1^,^2^. Despite the improvement of agricultural and manufacturing practices, mycotoxin contamination cannot be avoided and still represents a permanent health risk for both humans and animals. It is thus important to develop decontamination strategies^3^. Among mycotoxins, deoxynivalenol (DON) produced by *Fusarium* species, is commonly detected in cereal crops, including wheat, barley, and maize. It is the most abundant trichothecene in food with a frequent occurrence at toxicologically relevant concentrations worldwide^4^,^5^.

DON causes acute and chronic disorders in humans and animals, with the gastrointestinal tract being an organ sensitive to its adverse effects^6^. DON affects the intestinal histomorphology, impairs barrier function and nutrient absorption^7^,^8^. DON also disrupts the local intestinal immune response; it triggers and potentiates intestinal inflammation^9^,^10^. At the cellular and subcellular level, DON binds to the ribosome, inhibits protein and nucleic acid synthesis and triggers ribotoxic stress^11^,^12^,^13^ leading to the activation of kinases, MAPKs and their downstream signaling pathways^14^.

Several strategies have been developed to limit DON toxicity^15^, among them, bacterial biotransformation which depends on the ability of microorganisms to generate DON metabolites with reduced toxicity. De-epoxidation is a reductive chemical reaction opening the 12,13-epoxy ring transforming DON into its de-epoxide metabolite de-epoxy-deoxynivalenol (deepoxy-DON or DOM-1) (Supplementary Figure S1)^16^. Several microbial strains are capable of DON de-epoxidation^15^,^17^. Several *in vitro* studies demonstrated the reduced toxicity of deepoxy-DON. *In vivo* trials on farm animals receiving feed contaminated with DON have also shown a beneficial effect of the bacteria able to de-epoxidize DON, according to zootechnical parameters and immune response^18^. The hydroxyl on carbon 3 also seems to be significant for the toxic activity of DON and a detoxification strategy targeting this part of the C3-OH, leading to the formation of 3-epi-DON, was recently proposed^19^. Four bacterial strains, all isolated from soil, have been described to epimerize DON into 3-epi-DON^20^,^21^. Only one paper has investigated the effect of 3-epi-DON and demonstrated the lack of toxicity, both *in vitro* and *in vivo,* of this DON metabolite^22^.

The aim of the current study was to assess the efficacy of microbial transformation through analysis of the intestinal toxicity of deepoxy-DON and 3-epi-DON. Using physiological, histological and transcriptomic analysis, we have observed reduced toxicity of deepoxy-DON and 3-epi-DON, both for human intestinal epithelial cells and pig intestinal explants. We have further demonstrated that these microbial metabolites of DON fit into the ribosome pocket but do not elicit ribotoxic stress or activate the MAPKinase pathway. Our paper provides the first molecular insight for the reduced toxicity of deepoxy-DON and 3-epi-DON.

## Results

### Deepoxy-DON and 3-epi-DON do not impair proliferation of human intestinal cells

**Figure 1:**
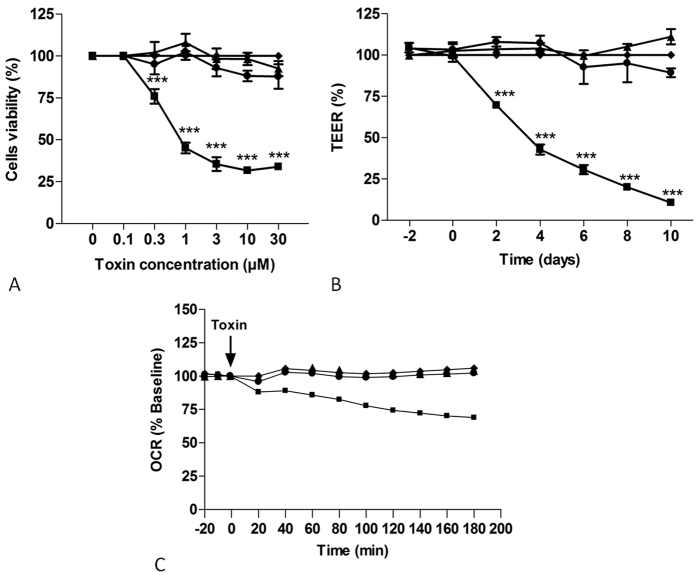
Effects of deepoxy-DON or 3-epi-DON on human intestinal epithelial cells. Activation of cytotoxicity (Panel A), TEER (Panel B) and OCR (Panel C). (Panel A): Proliferative Caco-2 cells were incubated with increasing concentrations of diluent (♦), DON (◾), deepoxy-DON (▴) or 3-epi-DON (●) for 48 hours. Cell viability evaluated by measurement of ATP, is expressed as % of control cells. (Panel B): Caco-2 cells, differentiated on inserts, were treated with 10 μM of diluent (♦), DON (◾), deepoxy-DON (▴) or 3-epi-DON (●) and TEER was measured. (Panel C): After establishment of baseline oxygen consumption rate in proliferated Caco-2 cells seeded to 1.5 × 10^4 ^cells/well, diluent (♦), DON (◾), deepoxy-DON (▴) or 3-epi-DON (●), was injected at final concentration of 10 μM as indicated by the arrow. The rate of oxygen consumption was then measured for the indicated time. For visual clarity, statistical indicators were omitted from the graph. The OCR values are shown as the percent of baseline for each group. Results are expressed as mean ± SEM of 3–4 independent experiments, ***p < 0.001.

Comparative effects of deepoxy-DON, 3-epi-DON and DON were first evaluated on proliferating human Caco-2 cells. The cell viability was assessed by the quantification of ATP using the luminescent cell viability assay. As shown in Fig. 1, Panel A, 48 hours exposure to deepoxy-DON or 3-epi-DON at concentrations up to 30 μM had no significant impact on cell viability. By contrast, DON markedly decreased the viability of proliferating cells in a dose-dependent manner; exposure to 10 μM of DON for 48 hours reduced cell viability by approximately 70%. The IC50 was calculated at 1.30 μM.

### Deepoxy-DON and 3-epi-DON do not impair cell viability and TEER of differentiated human intestinal cells

The comparative toxicity of deepoxy-DON, 3-epi-DON and DON was also performed on differentiated Caco-2 cells through the measurement of TEER. As already described^23^, differentiated cells are more resistant to DON and at least 30 μM are needed to induce a significant decrease in viability. At 10 μM of DON, a significant decrease of TEER in differentiated Caco-2 cells at a non-cytotoxic dose was observed (Fig. 1, Panel B). The decrease was time-dependent and reached 25% after 2 days and about 90% at 10 days. By contrast, cells treated with 10 μM deepoxy-DON or 3-epi-DON didn’t show any decrease in TEER.

### Deepoxy-DON and 3-epi-DON do not affect oxygen consumption in Caco-2 cells

The impact of deepoxy-DON, 3-epi-DON and DON on bioenergetic function in Caco-2 cells was evaluated using extracellular flux analyses. As shown in Fig. 1, Panel C, DON linearly decreased the rate of oxygen consumption in a time-dependent manner starting at the 40 minute stage. Approximately 3 hours after DON exposure, oxygen consumption values were 32% less than the base value. By contrast, deepoxy-DON and 3-epi-DON had no effect on the cellular oxygen consumption of proliferating Caco-2 cells and displayed bioenergetics profiles comparable to that of control cells.

### Deepoxy-DON and 3-epi-DON do not induce histological alterations of intestinal explants

**Figure 2:**
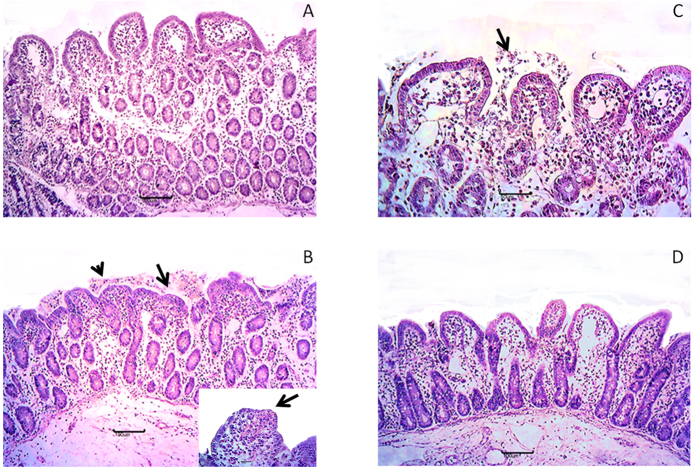
Comparative effects of deepoxy-DON and 3-epi-DON and DON on morphology of intestinal explants. Jejunal explants from 4 different animals were exposed for 4 hours, to diluent or 10 μM toxins and stained with HE for histological analysis. Normal villi lined with columnar enterocytes were observed on control explants (Panel A) multifocal villi atrophy (arrow) and cell debris (arrowhead), apical necrosis (insert) on DON explants (Panel B) histological aspects similar to control group on deepoxy-DON (Panel C) or 3-epi-DON (Panel D) explants. Bar 100 μm; insert bar 20 μm.

In order not to restrict the observations to an intestinal cell line, experiments were also performed on jejunal explants, a model developed to assess short-term effects of mycotoxins^24^. The effects on intestines of deepoxy-DON, 3-epi-DON and DON were first compared with histology (Fig. 2). Lymphatic vessel dilation was observed at different intensities in all groups. Control explants displayed normal villi lined with columnar enterocytes (Fig. 2, Panel A). Explants exposed to deepoxy-DON (Fig. 2, Panel C) and 3-epi-DON (Fig. 2, Panel D) presented similar features but mild interstitial edema and cell debris on apical surface (arrow) were also observed. By contrast, multifocal to diffuse villi atrophy, multifocal villi fusion (arrows), necrosis of apical enterocytes and cellular debris (arrowhead, Fig. 2, Panel B) were observed after 4 hours of explant incubation with 10 μM of DON.

### Deepoxy-DON and 3-epi-DON do not induce intestinal inflammation

To complete the analysis of the intestinal toxicity of deepoxy-DON and 3-epi-DON, their effects on the expression of inflammatory genes were analyzed by RT-qPCR. As already described^9^, a strong intestinal inflammatory response was observed in jejunal explants in the presence of DON and a significant increase in expression of IL-1α, TNFA, IL-1β, IL-8, IL-12p40, Il-17A and IL-22 was also observed (Table 1). By contrast, no induction in the expression of these genes was observed in deepoxy-DON and 3-epi-DON treated explants, demonstrating that microbial transformation of DON to deepoxy-DON or 3-epi-DON led to decreased inflammatory response in intestinal explants (Table 1).

### DON but not deepoxy-DON and 3-epi-DON changes gene expression profile in intestinal explants

**Figure 3:**
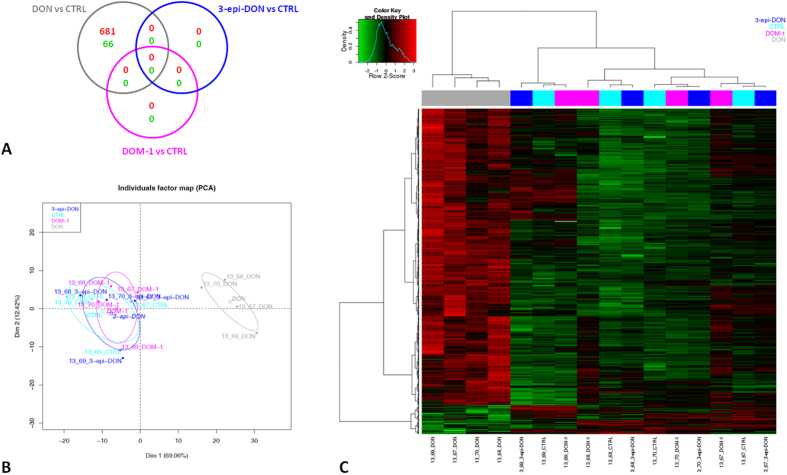
Gene expression profile of intestinal explants exposed to deepoxy-DON, 3-epi-DON or DON. Jejunal explants from 4 different animals were exposed for 4 hours, to diluent or 10 μM toxins and gene expression was analyzed with a 60 K microarray. (Panel A): Venn diagram illustrating the overlaps between the probes significantly up- or down-regulated in response to DON, deepoxy-DON (DOM-1) and 3-epi-DON treatment. (Panel B): Principal Component Analysis of differentially expressed probes between DON, deepoxy-DON (DOM-1), 3-epi-DON and control (747 with BH adjusted p-*value* < 0.05). (Panel C): Heat map representing differentially expressed probes between DON, deepoxy-DON (DOM-1), 3-epi-DON and control explant. Red and green colors indicate values above and below the mean (average Z-score) respectively. Black color indicates values close to the mean.

The effects of DON, deepoxy-DON and 3-epi-DON were investigated beyond the inflammatory response, through a genome wide transcriptomic analysis. Exposure to DON resulted in differential expression of 747 probes; 681 and 66 probes corresponding to 303 and 33 genes were up- and down-regulated respectively (Fig. 3, Panel A). By contrast, no genes were differentially expressed in deepoxy-DON and 3-epi-DON treated explants when compared to control explants, indicating that microbial transformation of DON to deepoxy-DON or 3-epi-DON abolishes the toxicity of the mycotoxin.

DON differentially expressed genes were then selected to perform principal component analysis (PCA) (Fig. 3, Panel B) and cluster analysis (Fig. 3, Panel C). Two clusters were distinguished, indicating up-regulated and down-regulated genes (Supplementary Table S2). The most significantly up-regulated genes in DON-treated explants, with a change of more than 2.4 fold compared to control, were immune genes such as *CCL20, CXCL2, PRDM1, AREG, CSF2, FOSL1* (Table 2). As expected, the 6 pro-inflammatory cytokines already tested in RT-qPCR analysis were also up-regulated in the DNA array analysis; a strong correlation between the two methods of analysis was observed (coefficient R^2^ = 0.96). DON also increased the expression of the ER heat shock protein HSP70 gene (*HSPA2)*, genes of ubiquitination pathway (*HSPA2, BIRC2, NEDD4L, BIRC3*) and genes of metallothioneins (*MT1A, MT1M* and *MT2B*). DON decreased expression of the *CHAC1* gene, genes for molecular transport including *ABCC2, SLC15A1, SLC9A2,* the *CCL24* gene which is thought to play a role in the immune response, the *MLEC* gene which is connected to protein misfolding under conditions of endoplasmic reticulum (ER) stress, and other genes (Table 2).

Pathway analysis of differentially expressed genes exposed to DON was performed using Ingenuity Pathway Analysis software (IPA). The top 10 scored pathways are listed in Table 3, the entire list can be found as Supplementary Table S3. DON disturbed pathways related to immunity/inflammation, such as cytokines regulations (IL-17 axis, IL-10 signaling), leukocytes functions (diapedesis), iNOS and NFkB signaling. DON affected other pathways associated with cell cycle regulation, apoptosis and ER stress response. Moreover, the results underlined the effects of DON on PXR/RXR, FXR/RXR signaling pathways and mitochondrial L-carnitine Shuttle Pathways.

### *In silico* analysis of the interaction of deepoxy-DON and 3-epi-DON with ribosomes and their inability to activate MAPKs

The above data indicate that microbial transformation of DON into deepoxy-DON or 3-epi-DON abrogates its toxicity. The last step of this study was to investigate the underlying mechanism and more specifically to determine the ability of DON, deepoxy-DON and 3-epi-DON to bind to the A site of the ribosome peptidyl transferase center and to activate the MAPKs.

**Figure 4:**
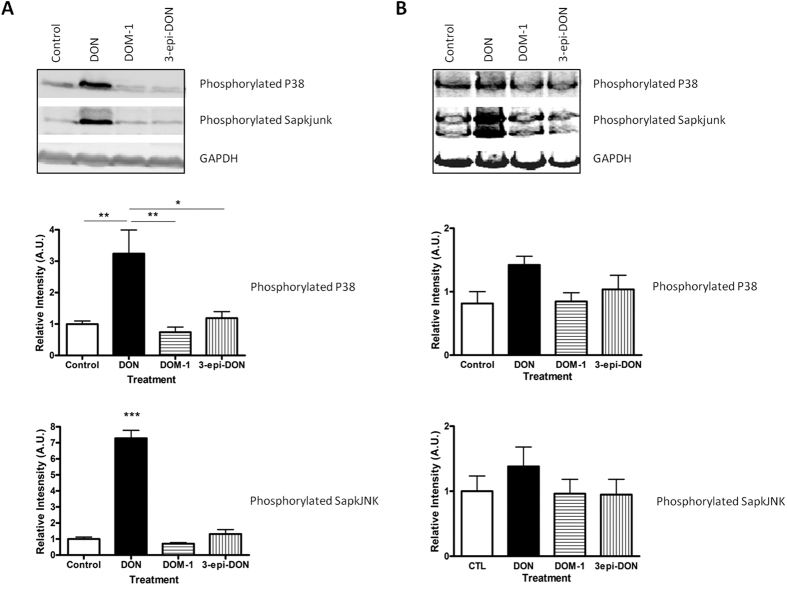
Effects of deepoxy-DON or 3-epi-DON on activation of MAPK on human intestinal epithelial cells. (Panel A): Caco-2 cells, differentiated on inserts. (Panel B): Jejunal explants. Samples were treated for 1 h with 10 μM toxins and analyzed by western blot for expression of phosphorylated P38, phosphorylated JNK and GAPDH, used as a protein loading control. Representative immunoblots and normalized expression graph. Results are expressed as mean ± SEM of 3–4 independent experiments, *p < 0.05, **p < 0.01, ***p < 0.001.

As expected, after 1 hour of exposure to 10 μM DON, MAPKs were activated in both differentiated monolayers Caco-2 cells and jejunal explants (Fig. 4). In cells, DON significantly increased phosphorylated p38 (3.24 ± 0.75 vs. 1 ± 0.09) compared to the control (relative intensity in arbitrary unit (A.U.); *p* < 0.05; n = 3) and phosphorylated SapKjunk (7.28 ± 0.64 vs. 1 ± 0.12 for control A.U.; p < 0.05; n = 3). By contrast, deepoxy-DON and 3-epi-DON were not able to activate these MAPKs in Caco-2 cells (Fig. 4 panel A). Similar trends were observed in jejunal explants (Fig. 4 panel B).

**Figure 5:**
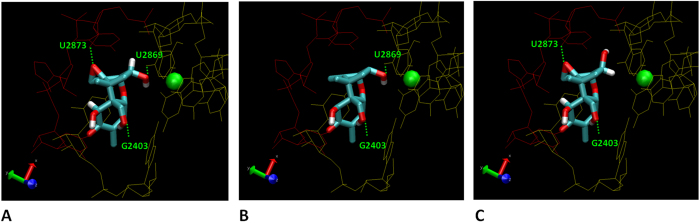
Interaction between the Ribosome 60S PTC subunit binding site and deepoxy-DON, 3-epi-DON or DON. Both sides of A site of the yeast ribosome 60S PTC subunit are colored in red and yellow respectively. Hydrogen and oxygen atoms are represented in white and red respectively. (Panel A): detailed views of the co-crystal (4UJX) of DON inside the A-site. (Panel B): detailed view of deepoxy-DON modeling inside the A-site. (Panel C): detailed views of 3-epi-DON modeling inside the A-site. The magnesium atom inside the A-site pocket has been pointed out in green.

The last step was to investigate the ability of DON and its bacterial metabolites, deepoxy-DON and 3-epi-DON, to bind to the 60S subunit inside the A-site of the peptidyl transferase center of the ribosome. The crystallographic data (4U53.pdb) obtained for DON and yeast ribosomes were used^13^. As shown in Fig. 5, (panel A) DON is able to fit in the pocket of the A-site of the ribosome 60S subunit. Within the pocket, the 3-hydroxyl group of DON is associated with a magnesium atom and stabilized by other nucleotides. In this position, DON forms 3 hydrogen bonds with the A-site. The first one is between the oxygen of the DON epoxy group on C12 and one hydrogen of the sugar of the uracil U2873; the second one is between the oxygen of the C15 group CH2OH and one hydrogen of the guanine basis G2403; and the last one is between the hydrogen of the C3 group and one oxygen of the uracil U2869. The *in silico* analysis revealed that both deepoxy-DON and 3-epi-DON were also able to fit into the pocket of the peptidyl transferase center of the ribosome (Fig. 5, Panel B and C). However, because of the absence of the epoxy group or the isomeric change, these two metabolites were only able to form 2 hydrogen bonds with the A sites of the peptidyl transferase center. Deepoxy-DON and 3-epi-DON did not form the bond with U2873 and U2869, respectively.

## Discussion

Despite good agricultural practices, contamination by mycotoxins cannot be avoided. Several strategies have been developed to reduce mycotoxin exposure. Among them, microbial transformation is of interest but requires demonstration of the absence of toxicity of the metabolites produced. The aims of the present study were (i) to analyze the intestinal toxicity of two bacterial metabolites of DON, deepoxy-DON and 3-epi-DON and (ii) to investigate the molecular basis for their reduced toxicity.

Through the action of bacteria, DON can be epimerized on the hydroxyl group of C3 or de-epoxidized on the C12-C13 epoxide^19^. Epimerization is an aerobic irreversible transformation that may require two enzymatic activities of partially overlapping substrate specificity which occur together in sequence: oxidation of DON to 3-keto-DON and then conversion to 3-epi-DON. To date only four bacterial strains have been described to epimerize DON to 3-epi-DON^19^. A very recent paper shows that this bacterial metabolite is substantially less toxic than DON when tested *in vitro* on proliferating human Caco2 cells, as well as *in vivo* when given orally to mice for 14 days^22^. Deepoxy-DON is obtained after de-epoxidation of DON; several bacterial species and enzymes are able to catalyze this reaction^19^. The lower toxicity of deepoxy-DON, as compared to DON has been demonstrated *in vitro* on Swiss mouse 3T3 fibroblasts^16^, lymphocytes^17^ and brine shrimp^25^. *In vivo* supplementation of DON-contaminated feed, with bacteria and/or an enzyme able to de-epoxidize DON, induced a reduction of the toxicity as shown by measurement of zootechnical or immune parameters^18^,^26^,^27^.

In the present study, we observed reduced intestinal toxicity of 3-epi-DON and deepoxy-DON when compared to DON. The toxicity of DON and its bacterial metabolites was first investigated on proliferating and differentiated Caco-2 cells. As already demonstrated, DON induced a significant decrease in Caco-2 cell proliferation, reduced their barrier function and altered their respiratory capacities^28^,^29^,^30^. This is the first investigation of the toxicity of deepoxy-DON on human intestinal epithelial cells, although the absence of toxicity of 3-epi-DON on Caco-2 cells has been recently demonstrated^22^.

The toxicity of DON and its bacterial metabolites on intestinal tissues was further evaluated. Because of the difficulties accessing human intestinal samples, the study was performed on porcine intestinal explants. Indeed, pigs are very sensitive to DON and can be considered good models for extrapolation to humans, with a digestive physiology very similar to that of humans^8^,^31^. Histological assessment showed normal villi lined with columnar enterocytes, mild interstitial edema and cell debris on the apical surface for the control, 3-epi-DON and deepoxy-DON treated explants. This is in accordance with the absence of histopathological lesions observed in mice after a 14 day oral exposure to 25 or 100 mg 3-epi-DON/Kg bw (body weight)^22^. Effects of purified deepoxy-DON on the intestine have never been tested, however nutritional strategies including bacteria/enzyme transforming DON to deepoxy-DON have reduced the occurrence and extent of intestinal lesions^18^ and showed the same zootechnical performance as in control animals^26^,^27^. By contrast, as already shown, treatment with 10 μM of DON induces intestinal damage indicated by villi atrophy and villi fusion^24^. To confirm that the two microbial transformation products of DON were not toxic, a pan-genomic analysis using a DNA array containing 62,976 probes was performed on jejunal explants. It revealed that no probes were differentially expressed between control explants and the ones treated with either deepoxy-DON or 3-epi-DON. To the best of our knowledge this is the first genome wide analysis performed for deepoxy-DON and 3-epi-DON.

The global transcriptomic analysis of the effect of DON on the intestine indicated that DON does not only interfere with genes involved in the immune response. As already described for human and murine thymus cells^32^,^33^,^34^, DON exposure also targets ER (endoplasmatic reticulum) stress, protein synthesis, oxidative stress, cell cycle regulation and apoptosis in intestinal tissues. The strong alteration of the gene *MLEC* implicated in misfolded glycoprotein quality control observed herein is likely due to the arrest of translation induced by ribotoxic stress. This leads to less protein entering the ER to temper the unfolded protein response and therefore protein synthesis^33^. The increased gene expression of the ER heat shock protein HSP70 could also reduce the accumulation of unfolded protein in ER lumen. An increased expression of some genes involved in the ubiquitination pathway was observed in the presence of DON. This result could indicate that the presence of DON may induce the increase in proteins involved in protein degradation^33^,^35^,^36^. Our data also underline the decrease of the unfolded protein response pro-apoptotic gene CHAC1. The CHAC1 protein seems to play a role in glutathione degradation^37^. ER stress could also induce leakage of calcium from the reticulum leading to activation of NFkB, NRF2-mediated oxidative stress response and apoptosis^33^. The present work emphasizes the effect of DON on metallothioneins MT1A, MT1M and MT2B. A relationship between metallothionein protein levels, used as a marker of oxidative stress, and mycotoxins in the liver of rats fed on naturally contaminated wheat has been reported^38^. Therefore, it could be assumed that MTs are associated with pathways protecting the intestine against DON toxicity. The present study underlines the effect of DON on the genes of intestinal transporters. DON decreases the expression of the solute carrier *SLC15A1* and *SLC9A2* involved in proton-coupled oligopeptides transporter PepT1 and a Na+/H+ exchanger, respectively^39^,^40^. Similar effects on other mRNA expression transporters as sugars transporters were described in the jejunum and to a lesser extent in the liver of broilers exposed to DON^41^. Accordingly, it has been experimentally shown that DON decreases the intestinal uptake of various nutrients in human epithelial intestinal cell line HT-29-D4^42^. This effect is likely due to a specific modulation of intestinal transporters expression, rather than a consequence of cell damage. The transcriptomic analysis demonstrates that DON down-regulates the expression of *ABCC2* gene that encodes for MRP2, a protein involved in efflux of DON and other mycotoxins and also in the transport of a wide range of organic anions including bile salt flow^43^. An action of DON on mitochondrial dysfunction, attested to by the down-regulation of CPT1A mRNA was also observed in this study. CPT1A encodes for a key regulatory enzyme of β-oxidation and is required for transport of long chain fatty acids into mitochondria^44^. The modulation of β-oxidation in addition to the modulation of intestinal transporters could explain the energy failure reported after DON exposure^42^. It is now necessary to investigate these changes at the protein level.

The use of bacteria is a promising approach to DON decontamination. In the present study we observed that deepoxy-DON and 3-epi-DON were devoid of intestinal toxicity. The underlying mechanism was further investigated. DON is known to develop its toxic potential by interacting with the peptidyl transferase at the 60S ribosomal subunit level, blocking the protein synthesis at the elongation step, inducing a ribotoxic stress and activating MAPKinases^7^,^12^,^13^. In accordance with literature, we observed that DON induced phosphorylation of JNK and p38 proteins^24^,^45^. By contrast, deepoxy-DON and 3-epi-DON did not active these signaling pathways, suggesting an absence of ribotoxic stress. To further the analysis, a modeling of deepoxy-DON and 3-epi-DON in the ribosome peptidyl transferase center was performed. DON and its bacterial metabolites fit into the A-site pocket, however whereas DON binds to the peptidyl transferase center with three hydrogen bonds, only two hydrogen bonds were identified between deepoxy-DON or 3-epi-DON and the peptidyl transferase center. This suggests that the absence of the epoxy group or the isomeric transformation decreases the affinity of these latter metabolites for the active site pocket A of the ribosome and prevents the induction of ribotoxic stress. *In silico* modeling revealed that a third hydrogen bond (the one between the oxygen of the C15 group CH2OH and the hydrogen of the guanine base G2403) could be involved in the interaction of DON with the ribosome. It would be of interest to establish whether this hydrogen bond is necessary for the toxicity of DON. Unfortunately we were not able to identify a proper DON metabolite or another fusariotoxin metabolite to confirm the involvement of this hydrogen bond in the structure-toxicity relationship. A more thorough analysis, with additional trichothecenes would be needed to fully determine the molecular basis of toxicity.

In conclusion, the present study confirms that the toxicity of DON is not only linked to the epoxy group but is also influenced by the C3 group^16^,^19^,^46^. It demonstrates that microbial biotransformation of DON into deepoxy-DON or 3-epi-DON decreases the intestinal toxicity of this mycotoxin. The underlying metabolism causes decreased affinity of the metabolites to the ribosome and the lack of MAPKinases activation. These data significantly increase the current knowledge of intestinal toxicity of DON, deepoxy-DON and 3-epi-DON and contribute to the evaluation of the effectiveness of the microbial biotransformation strategies in the fight against mycotoxins.

## Methods

### Toxins

Purified DON was purchased from Sigma-Aldrich (St Louis, MO, USA). Deepoxy-DON was obtained by transformation of crystalline DON (Romer Labs, Tulln, Austria), dissolved in medium 10^47^ at a concentration of 2 mg/ml, by inoculation with BBSH 797, *Gen. nov. sp. nov. of family Coriobacteriaceae* in sterile medium, at 37 °C for six days. Biotransformation of DON to deepoxy-DON was confirmed by LC-MS/MS, and deepoxy-DON was purified by solid phase extraction and preparative HPLC^48^. The purity of the deepoxy-DON preparation was 99%, based on chromatograms recorded at 220 nm. The identity of deepoxy-DON was confirmed by comparison of enhanced product ion spectra of the preparation used in the present study with a reference standard (Romer Labs, Tulln, Austria) recorded on a 4000 QTrap mass spectrometer at a collision energy of 30 eV (Supplementary Figure S2).

3-epi-DON was produced by microbial transformation of DON^21^. Briefly, DON was co-incubated with the bacterial strain, *Devosia mutans* 17-2-E-8, in corn meal broth medium at 28 °C for 48 h. High-speed counter-current chromatography (HSCCC) and preparative high performance liquid chromatography (prep-LC) were applied to separate 3-epi-DON. The obtained product was analyzed by liquid chromatography (LC) and identified by congruent retention time and UV/Vis spectrum and mass spectrometric (MS) data. Nuclear magnetic resonance (NMR) experiments such as correlation spectroscopy (COSY), heteronuclear single quantum coherence (HSQC) and nuclear overhauser effect (NOE) were conducted for structural characterization of 3-epi-DON. Purification and structure identification of 3-epi-DON was already demonstrated in a previous publication^21^. The 3-epi-DON used in the experiment had a purity of 96.8%.

Toxins were dissolved in dimethyl sulfoxide (DMSO) (Sigma-Aldrich) and stored at −20 °C until use.

### Caco-2 cell culture

Caco-2 cells (passages 99 - 106) obtained from the TC7 were cultured in 75-cm^2^ culture flasks (Cellstar cell culture flasks, Sigma-Aldrich) in Dulbecco’s Modified Eagle Medium enriched with glutamine (Gibco, Cergy-Pontoise, France), supplemented with 10% of heat inactivated fetal bovine serum, 0.5% of gentamycine (Eurobio, Courtaboeuf, France) and 1% of non-essential amino acids (Sigma-Aldrich). Cells were maintained at 37 °C in an atmosphere of 5% CO_2_ and 90% relative humidity. The medium was changed every 2 days. Cells were passaged once a week. The partially confluent cell monolayers were trypsinized with Trypsin-EDTA (Eurobio).

### Cell viability assay

Cell viability assay was performed with the CellTiter-Glo Luminescent Cell Viability Assay (Promega, Madison, USA) according to manufacturer’s instruction. This test measures the quantity of ATP, proportional to the quantity of cells. Cells were seeded at the density of 1.56 × 10^5 ^cells/cm^2^ in 96-well microtiter plates. Cells were grown for 24 hours and exposed to DON, deepoxy-DON or 3-epi-DON, or corresponding concentrations of DMSO, for 48 hours. Luminescence was measured with a spectrophotometer (TECAN Infinite M200, Männedorf, Switzerland).

### Trans-epithelial electrical resistant measurements

To assess the integrity of individual monolayers, trans-epithelial electrical resistance (TEER) was measured as already described^49^,^50^. Cells (1.34 × 10^5 ^cells/cm^2^) were grown until differentiation on polyethylene terephthalate membrane inserts (surface area 0.3 cm^2^, pore size 0.4 μm) in 24-well format (Becton Dickinson, Pont de Claix, France). The medium was changed every two days. Differentiated cells were exposed to 10 μM of diluent or toxins, DON, deepoxy-DON or 3-epi-DON. The culture medium in the apical side of differentiated cells in each well was replaced every two days with medium containing toxin. The TEER was measured for each well daily for 11 days using a Millicell-ERS Voltohmmeter (Millipore, Saint-Quentin-en-Yvelines, France). TEER values were expressed as % of initial values.

### Oxygen consumption measurements

The acute effect of toxins on oxygen consumption of Caco-2 cells was assessed using an XF24 extracellular flux analyzer (Seahorse Bioscience, North Billerica, USA). The procedure was performed according to the manufacturer’s instructions. Briefly, cells were cultured in XF24 cell culture microplates at 1.5 × 10^4^ cells per well and maintained as described above. Oxygen consumption rates (OCR) were measured in proliferated Caco-2 cells in non-buffered DMEM (Seahorse Bioscience) supplemented with 10 mM glucose, 2 mM sodium pyruvate (Sigma-Aldrich) and 2 mM glutamine (Eurobio) and adjusted to pH 7.4. OCR was monitored every 20 minutes before (basal level) and after injection of diluent or toxins (10 μM). OCR values from each well were normalized against viable cell counts (calculated with a Malassez cell) and expressed as a percentage of the baseline value.

### Intestinal jejunal explants

Jejunal explants were obtained from 5 week old crossbred castrated male piglets as described previously^24^. The experiment was conducted under the guidelines of the French Ministry of Agriculture for animal research. All animal experimentation procedures were approved by the Ethics committee of Pharmacology-Toxicology of Toulouse-Midi-Pyrénées in animal experimentation (Toxcométhique), N°: TOXCOM/0017/IO PP, in accordance with the European Directive on the protection of animals used for scientific purposes (Directive 2010/63/EU). Two authors (I.P.O. and A.P.) have an official agreement with the French Veterinary Services permitting animal experimentation. Explants were treated for 4 hours at 39 °C with 10 μM of toxins (DON, deepoxy-DON or 3-epi-DON) or diluent (DMSO) in complete medium. After incubation, treated explants were fixed in 10% formalin (Sigma-Aldrich) for histological analysis or stored at −80 °C for RNA extraction.

### Histological assessment

Explants fixed with 10% formalin for 24 hours were dehydrated and embedded in paraffin wax (Labonord, Templemars, France) according to standard histological procedures. Sections of 5 μm were stained with haematoxylin and eosin (Sigma-Aldrich) for histopathological assessment. Histological findings were scored based on histological changes and the severity of lesions as previously described^24^.

### Gene expression analysis of explants by RT-qPCR

For the gene expression analysis, total RNAs were extracted in lysing matrix D tubes (MP Biomedicals, Illkirch, France) containing guanidine thiocyanate-acid phenol (Eurobio). The quality of these RNA was assessed (Agilent RNA 6000 Nano Kit Quick, Agilent Bioanalyzer 2100); the mean RNA Integrity Number (RIN) of these mRNA preparations was 6.32 ± 0.83.

Reverse transcription and RT-qPCR steps were performed as already described^51^,^52^ with previously published primers (Supplementary Table S1)^23^. Amplification efficiency and initial fluorescence were determined by the ΔCt method. Obtained values were normalized using two reference genes, ribosomal protein L32 (RPL32) and cyclophilin A (CycloA). Gene expression levels of treated explants were expressed relative to the mean of the control explants.

### Gene expression analysis by microarray

The microarray GPL16524 (Agilent technology, 8 × 60 K) used in this experiment consisted of 43,603 spots derived from the 44 K (V2:026440 design) Agilent porcine specific microarray. This was enhanced with 9,532 genes from adipose tissue, 3,776 genes from the immune system and 3,768 genes from skeletal muscle^23^,^53^. For each sample, Cyanine-3 (Cy3) labeled cRNA was prepared from 200 μg of total RNA using the One-Color Quick Amp Labeling kit (Agilent) according to the manufacturer’s instructions, followed by Agencourt RNAClean XP (Agencourt Bioscience Corporation, Beverly, Massachusetts). About 600 ng of Cy3-labelled cRNA were hybridized on SurePrint G3 Porcine GE microarray (8×60 K) following the manufacturer’s instructions. Slides were scanned immediately, after washing on an Agilent G2505C Microarray Scanner with Agilent Scan Control A.8.5.1 software. All experimental details are available in the Gene Expression Omnibus (GEO) database under accession GSE66918 (http://www.ncbi.nlm.nih.gov/geo/query/acc.cgi?token=olixoose dvmdnqr&acc=GSE66918).

The differentially expressed (DE) genes (adjusted p-value ≤ 0.05) were hierarchically clustered and visualized in heat maps. Functional analysis of DE genes was performed using the Ingenuity pathway Analysis tool (IPA, http://www.ingenuity.com) to identify pathways and processes affected by toxins.

### Immunoblotting

Expression of the phosphorylated MAPK p38 and JNK (Jun amino-terminal kinases) was assessed on differentiated Caco-2 cells and jejunal explants by immunoblotting as previously described^49^,^54^. Cells differentiated on 24-well inserts or explants were treated with 10 μM of diluent (DMSO) or toxins, DON, deepoxy-DON or 3-epi-DON for 1 hour. Proteins were extracted, quantified and a total of 15 μg of protein was separated by SDS-PAGE. The membranes were probed with rabbit antibodies (Cell Signaling Technology, Danvers, USA) specific for: phospho-SPAK/JNK or phospho-p38 diluted at 1:500 or GAPDH diluted at 1:1000. After washing, the membranes were incubated with 1:10,000 CF^TM^770 goat anti-rabbit IgG (Biotium, Hayward, USA) for the detection. Antibody detection was performed using an Odyssey Infrared Imaging Scanner (Li-Cor Science Tec, les Ulis, France) with the 770 nm channel. The expression of the proteins was estimated after normalization with GAPDH signal.

### Molecular modeling

The yeast 80S ribosome X-ray structure, determined at a resolution of 3.3 Å (PDB code 4U53^13^), was used in all docking calculation. The whole chain A of the ribosome peptidyl transferase center was taken into account. The atomic coordinates of the PDB file were used for docking calculations using NAMD under VMD1.9^55^. VMD was used as visualization tools for various tasks (alignments, ligands location). The molecular structures of deepoxy-DON and 3-epi-DON were built using molefacture from the DON structure included in PDB-file 4U53, after adding hydrogen and after verification of the chirality and the type of each atoms. Ligand structures were minimized by NAMD using AMBER force field topology and parameter files. Molecular docking experiments of deepoxy-DON and 3-epi-DON were done in VMD based on the superposition of the carbon backbone of DON. To relax structures and to evaluate the interaction of hydrogen bonds between ribosome and ligand, a semi-rigid energy minimization was calculated by NAMD using AMBER topology and parameter files with a fixed ribosome structure and a flexible structure for the ligand. Associated to PDB-file 4U53 of DON, PDB-file 4U53 including structures of deepoxy-DON and 3-epi-DON are available in Supplementary Data (SupplementaryData_S1_4U53wDON, Supplementary Data_S2_4U53wdeepoxyDON and SupplementaryData_S3_4U53w3EpiDON).

### Statistical analysis

Data are expressed as a mean ± SEM of values. The results were analyzed using the Fisher test on equality of variances, one-way ANOVA and Bonferroni as a test post-hoc; p-values < 0.05 were considered significant. Microarray data from Feature Extraction software was analyzed with R using Bioconductor packages and the limma lmFit function as previously described^23^. Probes with adjusted p-value ≤ 0.05 were considered differentially expressed between treated and control conditions. Hierarchical clustering was applied to the samples and the probes using 1-Pearson correlation coefficient as distance and Ward’s criterion for agglomeration.

## Additional Information

**How to cite this article**: Pierron, A. *et al*. Microbial biotransformation of DON: molecular basis for reduced toxicity. *Sci. Rep.* **6**, 29105; doi: 10.1038/srep29105 (2016).

## Supplementary Material

Supplementary Information

Supplementary Dataset S1

Supplementary Dataset S2

Supplementary Dataset S3

## Figures and Tables

**Table 1:** DON but not deepoxy-DON & 3-epi-DON up-regulated mRNA relative expression levels of pro-inflammatory cytokines and chemokines in pig jejunal explants.

Cytokines	Explant treatments			
	Control	DON	Deepoxy-DON	3-epi-DON
*IL1B*	1.00 ± 0.40^a^	17.4 ± 5.1^b^	0.7 ± 0.2^a^	0.8 ± 0.3^a^
*IL1A*	1.00 ± 0.30^a^	3.9 ± 1.4^b^	0.9 ± 0.2^a^	0.9 ± 0.2^a^
*IL8*	1.00 ± 0.20^a^	4.5 ± 1.2^b^	1 ± 0.1a	0.9 ± 0.2^a^
*IL12p40*	1.00 ± 0.31^a^	2.3 ± 0.4^b^	1.2 ± 0.2^a^	0.9 ± 0.2^a^
*IL17A*	1.00 ± 0.50^a^	15.8 ± 5.6^b^	0.8 ± 0.1^a^	1.3 ± 0.4^a^
*IL22*	1.00 ± 0.30^a^	7.9 ± 1.3^b^	1.3 ± 0.5^a^	1.4 ± 0.5^a^
*TNFA*	1.00 ± 0.30^a^	3.5 ± 0.5^b^	1.1 ± 0.4^a^	1.1 ± 0.3^a^

*Notes:* results are expressed in arbitrary units relative to control group. Results are mean ± SEM of 6 animals. Means in a row without a common letter differ (Newman-Keuls test, *P* < 0.05).

**Table 2:** Top scored differentially expressed genes in DON treated porcine jejunal explants.

Gene symbol	Gene name	−log (p-value)	Ratio
a. Up-regulated genes			
IL1B	interleukin 1 beta	4.428	1.29E-11
CCL20	chemokine (C-C motif) ligand 20	3.481	1.79E-06
IL1A	interleukin 1. alpha	3.207	6.46E-09
CXCL2	chemokine (C-X-C motif) ligand 2	3.129	1.87E-04
IL22	interleukin 22	2.955	1.13E-07
PRDM1	PR domain containing 1 with ZNF domain	2.793	4.76E-06
AREG/AREGB	amphiregulin	2.662	1.94E-11
CSF2	colony stimulating factor 2 (granulocyte-macrophage)	2.593	1.86E-05
IL8	interleukin 8	2.585	1.25E-06
FOSL1	FOS-like antigen 1	2.447	4.22E-04
IER3	immediate early response 3	2.446	1.95E-04
CCR7	chemokine (C-C motif) receptor 7	2.325	1.79E-08
CALCB	calcitonin-related polypeptide beta	2.313	9.03E-11
GADD45A	growth arrest and DNA-damage-inducible alpha	2.270	5.61E-08
TNFAIP3	tumor necrosis factor alpha-induced protein 3	2.260	1.36E-08
RND1	Rho family GTPase 1	2.255	3.24E-06
IER2	immediate early response 2	2.227	3.44E-06
CD83	CD83 molecule	2.207	1.10E-05
PLAUR	plasminogen activator. urokinase receptor	2.085	9.86E-04
BTG2	BTG family member 2	2.073	1.25E-06
IFRD1	interferon-related developmental regulator 1	2.025	1.14E-08
RGS1	regulator of G-protein signaling 1	2.020	3.24E-06
GEM	GTP binding protein overexpressed in skeletal muscle	2.013	4.52E-05
CCL4	chemokine (C-C motif) ligand 4	2.004	6.44E-04
STX11	syntaxin 11	1.989	4.27E-05
GADD45G	growth arrest and DNA-damage-inducible gamma	1.881	2.26E-06
GADD45B	growth arrest and DNA-damage-inducible beta	1.873	9.01E-04
NEDD9	neural precursor cell expressed developmentally down-regulated 9	1.870	1.15E-10
LAMA3	laminin. alpha 3	1.858	2.07E-05
CD274	CD274 molecule	1.846	8.75E-11
IL17A	interleukin 17A	1.844	2.11E-11
b. Down-regulated genes			
* *CHAC1	cation transport regulator homolog 1 (*E. coli)*	−1.696	9.18E-04
* *ABCC2	ATP-binding cassette sub-family C (CFTR/MRP) member 2	−1.015	2.45E-06
* *SLC15A1	solute carrier family 15 (oligopeptide transporter) member 1	−0.851	3.56E-05
* *SLC9A2	solute carrier family 9 subfamily A (NHE2 cation proton antiporter 2) member 2	−0.804	9.09E-06
* *CCL24	chemokine (C-C motif) ligand 24	−0.784	8.75E-04
* *MTTP	microsomal triglyceride transfer protein	−0.755	3.26E-05
* *DMBT1	deleted in malignant brain tumors 1	−0.666	2.67E-04
* *MLEC	Malectin	−0.654	9.50E-04
* *SSH1	slingshot protein phosphatase 1	−0.628	1.06E-03
* *VPS26B	vacuolar protein sorting 26 homolog B (*S. pombe*)	−0.610	1.04E-03
* *ACE2	angiotensin I converting enzyme 2	−0.607	7.35E-04
* *SCGB2A1	secretoglobin. family 2A member 1	−0.594	2.74E-04
* *MYEOV	myeloma overexpressed	−0.592	1.58E-04
* *NPR3	natriuretic peptide receptor 3	−0.582	8.74E-04
* *CBL	Cbl proto-oncogene. E3 ubiquitin protein ligase	−0.574	3.70E-04
* *PLOD2	procollagen-lysine 2-oxoglutarate 5-dioxygenase 2	−0.547	3.98E-05
* *C4BPA	complement component 4 binding protein Alpha	−0.525	1.04E-03
* *ARHGEF37	Rho guanine nucleotide exchange factor (GEF) 37	−0.521	9.19E-04
* *DESI2	desumoylating isopeptidase 2	−0.501	3.04E-04
* *STOML3	stomatin (EPB72)-like 3	−0.487	8.33E-04
* *UNC119B	unc-119 homolog B (*C elegans*)	−0.467	2.42E-04
* *ZER1	zyg-11 related. cell cycle regulator	−0.455	4.92E-04
* *EGLN1	egl-9 family hypoxia-inducible factor 1	−0.443	2.26E-04
* *TCAP	titin-cap	−0.441	8.90E-04
* *PECAM1	platelet/endothelial cell adhesion molecule 1	−0.431	1.43E-04
* *ZCCHC14	zinc finger CCHC domain containing 14	−0.430	5.16E-04
* *GALNT4	polypeptide N-acetylgalactosaminyltransferase 4	−0.395	6.91E-04
* *ANKRD13A	ankyrin repeat domain 13A	−0.388	8.85E-04
* *UNC45A	unc-45 homolog A (C. elegans)	−0.377	7.64E-04
* *TPP1	tripeptidyl peptidase I	−0.375	5.37E-04
* *OSBPL7	oxysterol binding protein-like 7	−0.349	1.05E-03

**Table 3:** Ten top scored canonical pathways differentially regulated in 10 μM DON treated porcine jejuna explants and list of genes in each pathway.

Ingenuity Canonical Pathways	−log (p-value)	Ratio	Molecules
a. Up-regulated pathways			
* *Granulocyte Adhesion and Diapedesis	1.18E01	1.1E-01	*CCL3,IL1B,MMP12,EZR,CCL20,CLDN4,CCL3L1/CCL3L3,SELE,MMP13,CXCL2,VCAM1,CXCL8, CXCR4, IL18, IL1RN,TNF,CXCR2,ICCL4,XCL1*
* *Agranulocyte Adhesion and Diapedesis	1.13E01	1.04E-01	*CCL3,IL1B,MMP12,EZR,CCL20,CLDN4,CCL3L1/CCL3L3,SELE,MMP13,CXCL2,VCAM1,CXCL8, CXCR4,IL18,IL1RN,TNF,CXCR2,IL1A,CCL4,XCL1*
* *Glucocorticoid Receptor Signaling	1.11E01	7.69E-02	*HSPA2,NFKB1,CCL3,CSF2,JAK2,IL1B,PLAU,SELE,NFKBIE,NFATC1,NFKBIA,VCAM1,CXCL8, FOS,IL1RN,DUSP1,SGK1,NR3C1,TNF,SMAD3,CDKN1A,IL10,FOXO3*
* *Differential Regulation of Cytokine Production in Intestinal Epithelial Cells by IL-17A and IL-17F	1.07E01	3.91E-01	*CCL3,CSF2,IL1B,TNF,IL1A,IL17A,IL10,CCL4,IL17F*
* *Communication between Innate and Adaptive Immune Cells	1.01E01	1.25E-01	*CCR7,CCL3,CSF2,IL1B,CCL3L1/CCL3L3,CD40,CXCL8,CD83,IL18,IL1RN,TNF,IL1A,IL10,CCL4*
* *Differential Regulation of Cytokine Production in Macrophages and T Helper Cells by IL-17A and IL-17F	1.01E01	4.44E-01	*CCL3,CSF2,IL1B,TNF,IL17A,IL10,CCL4,IL17F*
* *Hepatic Fibrosis/Hepatic Stellate Cell Activation	9.55E00	1.03E-01	*CCR7,NFKB1,IL1B,EDNRB,MMP13,CD40,VCAM1,CXCL8,IFNGR1,TNF,SMAD3,IL1A,EDN1,IL4R, IL10,TIMP*
* *IL-10 Signaling	9.48E00	1.54E-01	*FOS,IL18,NFKB1,IL1RN,IL1B,SOCS3,TNF,IL1A,IL4R,IL10,NFKBIE,NFKBIA*
* *T Helper Cell Differentiation	9.41E00	1.67E-01	*GATA3,IFNGR1,BCL6,IL18,STAT4,TNF,ICOSLG/LOC102723996,IL17A,IL4R,IL10,CD40,IL17F*
* *Atherosclerosis Signaling	9.4E00	1.08E-01	*NFKB1,IL1B,SELE,MMP13,CD40,VCAM1,CXCL8,CXCR4,IL18,IL1RN,TNF,IL1A,F3,TNFRSF12A, PLA2G4A*
b. Down-regulated pathways			
* *PXR/RXR Activation	2.28E00	2.17E-02	*ABCC2, CPT1A*
* *FXR/RXR Activation	2.07E00	1.82E-02	*ABCC2, MTTP*
* *Mitochondrial L-carnitine Shuttle Pathway	1.57E00	4.55E-02	*CPT1A*
* *Granulocyte Adhesion and Diapedesis	1.49E00	1.1E-02	*CCL24, PECAM1*
* *Agranulocyte Adhesion and Diapedesis	1.44E00	1.04E-02	*CCL24, PECAM1*
* *LPS/IL-1 Mediated Inhibition of RXR Function	1.31E00	8.16E-03	*ABCC2, CPT1A*
* *Complement System	1.28E00	2.86E-02	*C4BPA*
* *Erythropoietin Signaling	9.87E-01	1.27E-02	*CBL*
* *Chemokine Signaling	9.75E-01	1.33E-02	*CCL24*
* *Ephrin B Signaling	9.46E-01	1.22E-02	*CBL*

## References

[b1] Bennett J. W. & Klich M. Mycotoxins. Clin Microbiol Rev 16, 497 (2003). 12857779 10.1128/CMR.16.3.497-516.2003 PMC164220

[b2] Frisvad J. C., Nielsen K. F. & Samson R. A. Recommendations concerning the chronic problem of misidentification of mycotoxigenic fungi associated with foods and feeds. Adv Exp Med Biol 571, 33 (2006). 16408592 10.1007/0-387-28391-9_2

[b3] Awad W. A., Ghareeb K., Bohm J. & Zentek J. Decontamination and detoxification strategies for the Fusarium mycotoxin deoxynivalenol in animal feed and the effectiveness of microbial biodegradation. Food Addit Contam Part A Chem Anal Control Expo Risk Assess 27, 510 (2010). 20234966 10.1080/19440040903571747

[b4] EFSA, Deoxynivalenol in food and feed: occurrence and exposure. EFSA Journal 11, 3379 (2013).

[b5] CAST, Mycotoxins: Risks in Plant, Animal, and Human Systems. Council for Agricultural Science and Technology-Potential economic costs of mycotoxins in United States Task Force Rep. 138 (Iowa, USA), 136 (2003).

[b6] Pestka J. J. Deoxynivalenol: mechanisms of action, human exposure, and toxicological relevance. Arch Toxicol 84, 663 (2010). 20798930 10.1007/s00204-010-0579-8

[b7] Maresca M. From the gut to the brain: journey and pathophysiological effects of the food-associated mycotoxin Deoxynivalenol. Toxins 5, 784 (2013). 23612752 10.3390/toxins5040784 PMC3705292

[b8] Pinton P. & Oswald I. P. Effect of deoxynivalenol and other Type B trichothecenes on the intestine: a review. Toxins 6, 1615 (2014). 24859243 10.3390/toxins6051615 PMC4052256

[b9] Cano P. M. . Deoxynivalenol as a new factor in the persistence of intestinal inflammatory diseases: an emerging hypothesis through possible modulation of Th17-mediated response. PLoS One 8, e53647 (2013). 23326479 10.1371/journal.pone.0053647 PMC3542340

[b10] Vandenbroucke V. . The mycotoxin deoxynivalenol potentiates intestinal inflammation by salmonella typhimurium in porcine ileal loops. PLoS One 6, e23871 (2011). 21909370 10.1371/journal.pone.0023871 PMC3166085

[b11] Shifrin V. I. & Anderson P. Trichothecene mycotoxins trigger a ribotoxic stress response that activates c-Jun N-terminal kinase and p38 mitogen-activated protein kinase and induces apoptosis. J Biol Chem 274, 13985 (1999). 10318810 10.1074/jbc.274.20.13985

[b12] Pestka J. J., Zhou H. R., Moon Y. & Chung Y. J. Cellular and molecular mechanisms for immune modulation by deoxynivalenol and other trichothecenes: unraveling a paradox. Toxicol Lett 153, 61 (2004). 15342082 10.1016/j.toxlet.2004.04.023

[b13] Garreau de Loubresse N. . Structural basis for the inhibition of the eukaryotic ribosome. Nature 513, 517 (2014). 25209664 10.1038/nature13737

[b14] Pestka J. J. Deoxynivalenol-induced proinflammatory gene expression: mechanisms and pathological sequelae. Toxins 2, 1300 (2010). 22069639 10.3390/toxins2061300 PMC3153246

[b15] Zhou T., He J. & Gong J. Microbial transformation of trichothecene mycotoxins. World Mycotoxin J 1, 23 (2008).

[b16] Sundstol Eriksen G., Pettersson H. & Lundh T. Comparative cytotoxicity of deoxynivalenol, nivalenol, their acetylated derivatives and de-epoxy metabolites. Food Chem Toxicol 42, 619 (2004). 15019186 10.1016/j.fct.2003.11.006

[b17] Schatzmayr G. . Microbiologicals for deactivating mycotoxins. Mol Nutr Food Res 50, 543 (2006). 16715543 10.1002/mnfr.200500181

[b18] Grenier B. . Biotransformation approaches to alleviate the effects induced by fusarium mycotoxins in swine. J Agric Food Chem 61, 6711 (2013). 23758213 10.1021/jf400213q

[b19] Karlovsky P. Biological detoxification of the mycotoxin deoxynivalenol and its use in genetically engineered crops and feed additives. Appl Microbiol Biotechnol 91, 491 (2011). 21691789 10.1007/s00253-011-3401-5 PMC3136691

[b20] Berthiller F. . Masked mycotoxins: a review. Mol Nutr Food Res 57, 165 (2013). 23047235 10.1002/mnfr.201100764 PMC3561696

[b21] He J. W. . An epimer of deoxynivalenol: purification and structure identification of 3-epi-deoxynivalenol. Food Addit Contam Part A Chem Anal Control Expo Risk Assess 32, 1523 (2015). 26247304 10.1080/19440049.2015.1072771

[b22] He J. W. . Toxicology of 3-epi-deoxynivalenol, a deoxynivalenol-transformation product by Devosia mutans 17-2-E-8. Food Chem Toxicol 84, 250 (2015). 26363308 10.1016/j.fct.2015.09.003

[b23] Pierron A. . Intestinal toxicity of the masked mycotoxin deoxynivalenol-3-beta-D-glucoside. Arch Toxicol in press (2016). 10.1007/s00204-015-1592-8 26404761

[b24] Lucioli J. . The food contaminant deoxynivalenol activates the mitogen activated protein kinases in the intestine: Interest of *ex vivo* models as an alternative to in vivo experiments. Toxicon 66, 31 (2013). 23403092 10.1016/j.toxicon.2013.01.024

[b25] Swanson S. P., Rood H. D. Jr., Behrens J. C. & Sanders P. E. Preparation and characterization of the deepoxy trichothecenes: deepoxy HT-2, deepoxy T-2 triol, deepoxy T-2 tetraol, deepoxy 15-monoacetoxyscirpenol, and deepoxy scirpentriol. Appl Environ Microbiol 53, 2821 (1987). 3435145 10.1128/aem.53.12.2821-2826.1987 PMC204205

[b26] Li X. Z. . Efficacy of detoxification of deoxynivalenol-contaminated corn by Bacillus sp. LS100 in reducing the adverse effects of the mycotoxin on swine growth performance. Food Addit Contam Part A Chem Anal Control Expo Risk Assess 28, 894 (2011). 21614709 10.1080/19440049.2011.576402

[b27] He P., Young L. G. & Forsberg C. Microbially detoxified vomitoxin-contaminated corn for young pigs. J Anim Sci 71, 963 (1993). 8478296 10.2527/1993.714963x

[b28] Alassane-Kpembi I. . New insights into mycotoxin mixtures: The toxicity of low doses of Type B trichothecenes on intestinal epithelial cells is synergistic. Toxicol Appl Pharmacol 272, 191 (2013). 23735874 10.1016/j.taap.2013.05.023

[b29] Akbari P. . Deoxynivalenol: a trigger for intestinal integrity breakdown. FASEB J 28, 2414 (2014). 24568843 10.1096/fj.13-238717

[b30] Bin-Umer M. A. . Elimination of damaged mitochondria through mitophagy reduces mitochondrial oxidative stress and increases tolerance to trichothecenes. Proc Natl Acad Sci USA 111, 11798 (2014). 25071194 10.1073/pnas.1403145111 PMC4136610

[b31] Nejdfors P., Ekelund M., Jeppsson B. & Westrom B. R. Mucosal in vitro permeability in the intestinal tract of the pig, the rat, and man: species- and region-related differences. Scand J Gastroenterol 35, 501 (2000). 10868453 10.1080/003655200750023769

[b32] van Kol S. W., Hendriksen P. J., van Loveren H. & Peijnenburg A. The effects of deoxynivalenol on gene expression in the murine thymus. Toxicol Appl Pharmacol 250, 299 (2011). 21074547 10.1016/j.taap.2010.11.001

[b33] Katika M. R. . Transcriptome analysis of the human T lymphocyte cell line Jurkat and human peripheral blood mononuclear cells exposed to deoxynivalenol (DON): New mechanistic insights. Toxicol Appl Pharmacol 264, 51 (2012). 22846391 10.1016/j.taap.2012.07.017

[b34] Mishra Sakshi, Dwivedi, Premendra D., Pandey, Haushila P. & Das Mukul Role of oxidative stress in Deoxynivalenol induced toxicity. Food Chem Toxicol 72, 20 (2014). 25010452 10.1016/j.fct.2014.06.027

[b35] Shen Y. . ER stress differentially regulates the stabilities of ERAD ubiquitin ligases and their substrates. Biochem Biophys Res Commun 352, 919 (2007). 17157811 10.1016/j.bbrc.2006.11.121

[b36] Osman A. M. . Protein expression profiling of mouse thymoma cells upon exposure to the trichothecene deoxynivalenol (DON): implications for its mechanism of action. J Immunotoxicol 7, 147 (2010). 20672443

[b37] Kumar A. . Mammalian proapoptotic factor ChaC1 and its homologues function as gamma-glutamyl cyclotransferases acting specifically on glutathione. EMBO Rep 13, 1095 (2012). 23070364 10.1038/embor.2012.156 PMC3512401

[b38] Vasatkova A. . Changes in metallothionein level in rat hepatic tissue after administration of natural mouldy wheat. Int J Mol Sci 10, 1138 (2009). 19399242 10.3390/ijms10031138 PMC2672023

[b39] Bookstein C. . Tissue distribution of Na+/H+ exchanger isoforms NHE2 and NHE4 in rat intestine and kidney. Am J Physiol 273, C1496 (1997). 9374634 10.1152/ajpcell.1997.273.5.C1496

[b40] Smith D. E., Clemencon B. & Hediger M. A. Proton-coupled oligopeptide transporter family SLC15: physiological, pharmacological and pathological implications. Mol Aspects Med 34, 323 (2013). 23506874 10.1016/j.mam.2012.11.003 PMC3602806

[b41] Dietrich B., Neuenschwander S., Bucher B. & Wenk C. Fusarium mycotoxin-contaminated wheat containing deoxynivalenol alters the gene expression in the liver and the jejunum of broilers. Animal 6, 278 (2012). 22436186 10.1017/S1751731111001601

[b42] Maresca M., Mahfoud R., Garmy N. & Fantini J. The mycotoxin deoxynivalenol affects nutrient absorption in human intestinal epithelial cells. J Nutr 132, 2723 (2002). 12221236 10.1093/jn/132.9.2723

[b43] Videmann B., Tep J., Cavret S. & Lecoeur S., Epithelial transport of deoxynivalenol: involvement of human P-glycoprotein (ABCB1) and multidrug resistance-associated protein 2 (ABCC2). Food Chem Toxicol 45, 1938 (2007). 17543436 10.1016/j.fct.2007.04.011

[b44] Nakamura M. T., Yudell B. E. & Loor J. J. Regulation of energy metabolism by long-chain fatty acids. Prog Lipid Res 53, 124 (2014). 24362249 10.1016/j.plipres.2013.12.001

[b45] Sergent T. . Deoxynivalenol transport across human intestinal Caco-2 cells and its effects on cellular metabolism at realistic intestinal concentrations. Toxicol Lett 164, 167 (2006). 16442754 10.1016/j.toxlet.2005.12.006

[b46] Sato I. & Ueno Y. Comparative toxicities of trichothecenes. In Rodricks J. V., Hesseltine C. W., Mehlman M. A. (eds) Mycotoxins in human and animal health Pathotox Publishers, Park Forest South, IL, 295 (1977).

[b47] Caldwell D. R. & Bryant M. P. Medium without rumen fluid for nonselective enumeration and isolation of rumen bacteria. Appl Microbiol 14, 794 (1966). 5970467 10.1128/am.14.5.794-801.1966 PMC1058417

[b48] Schwartz-Zimmermann H. E. . Deoxynivalenol (DON) sulfonates as major DON metabolites in rats: from identification to biomarker method development, validation and application. Anal Bioanal Chem 406(30), 7911 (2014). 25338936 10.1007/s00216-014-8252-3

[b49] Pinton P. . Toxicity of deoxynivalenol and its acetylated derivatives on the intestine: differential effects on morphology, barrier function, tight junction proteins, and mitogen-activated protein kinases. Toxicol Sci 130, 180 (2012). 22859312 10.1093/toxsci/kfs239

[b50] Loiseau N. . Fumonisin B_1_ exposure and its selective effect on porcine jenunal segment: sphingolipids, glycolipids and transepithelial-passage disturbance. Biochem. Pharmacol. 74, 144 (2007). 17499218 10.1016/j.bcp.2007.03.031

[b51] Halloy D. J., Gustin P. G., Bouhet S. & Oswald I. P. Oral exposure to culture material extract containing fumonisins predisposes swine to the development of pneumonitis caused by Pasteurella multocida. Toxicology 213, 34 (2005). 15979225 10.1016/j.tox.2005.05.012

[b52] Gourbeyre P. . Pattern recognition receptors in the gut: analysis of their expression along the intestinal tract and the crypt/villus axis. Physiol Rep 3(2), e12225 (2015). 25677543 10.14814/phy2.12225 PMC4393184

[b53] Gao Y. . Transcriptome analysis of porcine PBMC after stimulation by LPS or PMA/ionomycin using an expression array targeting the pig immune response. BMC genomic 11, 292 (2010). 10.1186/1471-2164-11-292 PMC2881026 20459780

[b54] Meissonnier G. M. . Subclinical doses of T-2 toxin impair acquired immune response and liver cytochrome P450 in pigs. Toxicology. 247, 46 (2008). 18355953 10.1016/j.tox.2008.02.003

[b55] Humphrey W., Dalke A. & Schulten K. VMD: visual molecular dynamics. J Mol Graph 14, 33 (1996). 8744570 10.1016/0263-7855(96)00018-5

