# Supplementary material for: Microbial biotransformation of DON: molecular basis for reduced toxicity
Source: Sci Rep. 2016 Jul 6;6:29105. doi: 10.1038/srep29105 (PMC4933977; doi:10.1038/srep29105)
Supplement: Supplementary Information [file srep29105-s1.pdf]

Microbial biotransformation of DON: molecular basis for reduced toxicity

Alix Pierron[1,2,3*] and Sabria Mimoun[1,2*], Leticia S. Murate[1,4], Nicolas Loiseau[1,2], Yannick Lippi[1,2], Ana-Paula F.L. Bracarense[4], Gerd Schatzmayr[3], Jian Wei He[5], Ting Zhou[5], Wulf-Dieter Moll[3] and Isabelle P. Oswald[1,2]

[1] Toxalim, Research Center in Food Toxicology, Université de Toulouse, INRA, UMR 1331, ENVT, INP-Purpan Toulouse, France

[2] Université de Toulouse, INP, UMR 1331, Toxalim, Toulouse, France

[3] BIOMIN Research Center Technopark 1, 3430 Tulln, Austria

[4] Universidade Estadual de Londrina, Lab. Patologia Animal, CP 6001, Londrina, Paraná, Brazil

[5] Guelph Food Research Center Agriculture & Agri-Food Canada, Guelph, Ontario N1G 5C, Canada

[*] equally contributed

Address correspondence to

Dr. Isabelle P. Oswald

INRA, UMR-1331, Toxalim, 180 chemin de Tournefeuille, 31027 Toulouse cedex 3, France

Phone +33 582066366

E-Mail: Isabelle.Oswald@toulouse.inra.fr

Supplementary materials

Supplementary Figure S1. Molecular structures of DON, deepoxy-DON and 3-epi-DON.

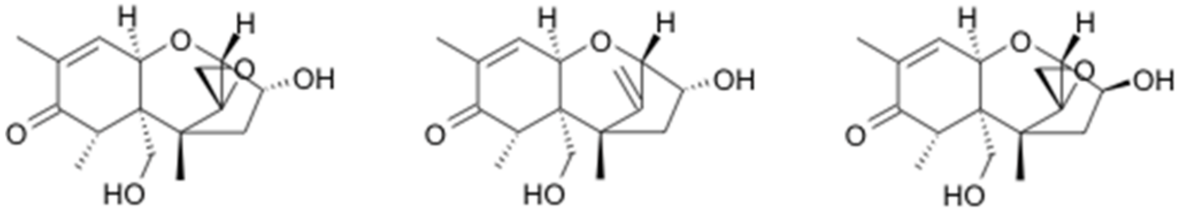

DON **deepoxy-DON** **3-epi-DON**

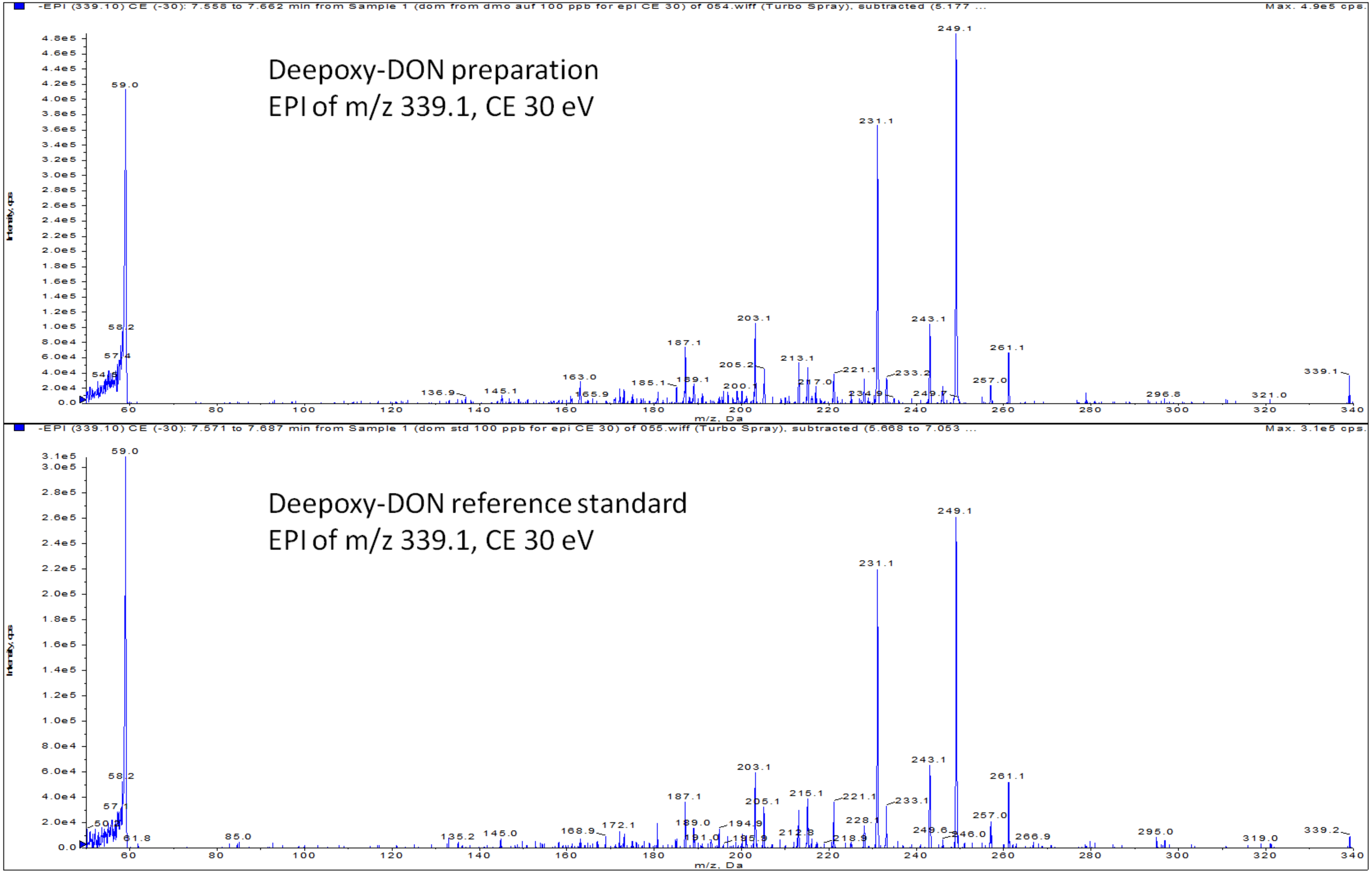
-EPI (339.10) CE (-30): 7.558 to 7.662 min from Sample 1 (dom from dmo auf 100 ppb for epi CE 30) of 054.wiff (Turbo Spray), subtracted (5.177 ...
Max. 4.9e5 cps.
Deepoxy-DON preparation
EPI of m/z 339.1, CE 30 eV
Intensity, cps
m/z, Da
249.1
231.1
59.0
203.1
243.1
187.1
261.1
213.1
205.2
221.1
233.2
339.1
-EPI (339.10) CE (-30): 7.571 to 7.687 min from Sample 1 (dom std 100 ppb for epi CE 30) of 055.wiff (Turbo Spray), subtracted (5.668 to 7.053 ...
Max. 3.1e5 cps.
Deepoxy-DON reference standard
EPI of m/z 339.1, CE 30 eV
Intensity, cps
m/z, Da
59.0
249.1
231.1
243.1
203.1
261.1
187.1
215.1
221.1
233.1
257.0
339.2

Supplementary Figure S2. Enhanced product ion spectra of parent ion [Deepoxy-DON + acetate]- (m/z = 339.1) recorded on a 4000 QTrap mass spectrometer at a collision energy of 30 eV. Top: our preparation used for the experiments; bottom: reference standard (Romer Labs, Tulln, Austria).

Supplementary Table S1. Primer sequences used for RT-qPCR analysis (F: Forward; R: Reverse)

Gene symbol	Gene name	Primer sequence	References
CycloA	Cyclophilin A	F: CCCACCGTCTTCTTCGACAT	NM_214353
		R: TCTGCTGTCTTTGGAACTTTGTCT	Gourbeyre et al. 2015
RPL32	Ribosomal Protein L32	F: AGTTCATCCGGCACCAGTCA	NM_001001636
		R: GAACCTTCTCCGCACCCTGT	Gourbeyre et al. 2015
IL1A	Interleukin 1- alpha	F: TCAGCCGCCCATCCA	NM_214029,1
		R: AGCCCCCGGTGCCATGT	Cano et al. 2013
IL1B	Interleukin 1 - beta	F: ATGCTGAAGGCTCTCCACCTC	NM_214055
		R: TTGTTGCTATCATCTCCTTGCAC	Gourbeyre et al. 2015
IL8	Interleukin - 8	F: GCTCTCTGTGAGGCTGCAGTTC	NM_213867
		R: AAGGTGTGGAATGCGTATTTATGC	Grenier et al. 2011
TNFA	Tumor necrosis factor -alpha	F: ACTGCACTTCGAGGTTATCGG	NM_214022
		R: GGCGACGGGCTTATCTGA	Gourbeyre et al. 2015
IL12p40	Interleukin 12 - p 40	F: GGTTTCAGACCCGACGAACTCT	NM_214013
		R : CATATGGCCACAATGGGAGATG	Cano et al., 2013
IL17A	Interleukin 17 - alpha	F: CCAGACGGCCCTCAGATTAC	AB102693
		R: CACTTGGCCTCCCAGATCAC	Cano et al. 2013
IL22	Interleukin - 22	F: AAGCAGGTCCTGAACTTCAC	AY937228
		R: CACCCTTAATACGGCATTGG	Cano et al. 2013

Supplementary Table S2: Complete list of differentially expressed genes in DON treated intestinal explants

Gene symbol	Gene name	-log (p-value)	Ratio
Up- regulated genes			

IL1B	interleukin 1. beta	4.428	1.29E-11
CCL20	chemokine (C-C motif) ligand 20	3.481	1.79E-06
IL1A	interleukin 1. alpha	3.207	6.46E-09
CXCL2	chemokine (C-X-C motif) ligand 2	3.129	1.87E-04
IL22	interleukin 22	2.955	1.13E-07
PRDM1	PR domain containing 1. with ZNF domain	2.793	4.76E-06
AREG/AREGB	amphiregulin	2.662	1.94E-11
CSF2	colony stimulating factor 2 (granulocyte-macrophage)	2.593	1.86E-05
IL8	interleukin 8	2.585	1.25E-06
FOSL1	FOS-like antigen 1	2.447	4.22E-04
IER3	immediate early response 3	2.446	1.95E-04
CCR7	chemokine (C-C motif) receptor 7	2.325	1.79E-08
CALCB	calcitonin-related polypeptide beta	2.313	9.03E-11
GADD45A	growth arrest and DNA-damage-inducible. alpha	2.270	5.61E-08
TNFAIP3	tumor necrosis factor. alpha-induced protein 3	2.260	1.36E-08
RND1	Rho family GTPase 1	2.255	3.24E-06
IER2	immediate early response 2	2.227	3.44E-06
CD83	CD83 molecule	2.207	1.10E-05
PLAUR	plasminogen activator. urokinase receptor	2.085	9.86E-04
BTG2	BTG family. member 2	2.073	1.25E-06
IFRD1	interferon-related developmental regulator 1	2.025	1.14E-08
RGS1	regulator of G-protein signaling 1	2.020	3.24E-06
GEM	GTP binding protein overexpressed in skeletal muscle	2.013	4.52E-05
CCL4	chemokine (C-C motif) ligand 4	2.004	6.44E-04
STX11	syntaxin 11	1.989	4.27E-05
GADD45G	growth arrest and DNA-damage-inducible. gamma	1.881	2.26E-06
GADD45B	growth arrest and DNA-damage-inducible. beta	1.873	9.01E-04
NEDD9	neural precursor cell expressed. developmentally down-regulated 9	1.870	1.15E-10
LAMA3	laminin. alpha 3	1.858	2.07E-05
CD274	CD274 molecule	1.846	8.75E-11
IL17A	interleukin 17A	1.844	2.11E-11
NFKBIA	nuclear factor of kappa light polypeptide gene enhancer in B-cells inhibitor. alpha	1.779	3.53E-05
SOCS3	suppressor of cytokine signaling 3	1.771	2.54E-06
F3	coagulation factor III (thromboplastin. tissue factor)	1.754	4.29E-05
IDO1	indoleamine 2.3-dioxygenase 1	1.748	2.92E-07
HAMP	hepcidin antimicrobial peptide	1.724	7.77E-07
SPRY2	sprouty homolog 2 (Drosophila)	1.723	1.83E-07

PHLDA1	pleckstrin homology-like domain. family A. member 1	1.708	1.57E-06
NABP1	nucleic acid binding protein 1	1.681	3.78E-07
BCL2A1	BCL2-related protein A1	1.681	5.18E-08
GPR65	G protein-coupled receptor 65	1.677	5.79E-10
TBC1D4	TBC1 domain family. member 4	1.652	2.82E-08
NFKBIZ	nuclear factor of kappa light polypeptide gene enhancer in B-cells inhibitor. zeta	1.652	6.58E-06
ADAMTS1	ADAM metallopeptidase with thrombospondin type 1 motif. 1	1.652	5.54E-06
TNF	tumor necrosis factor	1.647	4.65E-06
GPR183	G protein-coupled receptor 183	1.609	6.59E-07
ENC1	ectodermal-neural cortex 1 (with BTB domain)	1.602	3.23E-05
BIRC3	baculoviral IAP repeat containing 3	1.596	2.03E-07
IL17F	interleukin 17F	1.527	5.39E-09
RCAN1	regulator of calcineurin 1	1.524	2.18E-04
SELE	selectin E	1.521	2.09E-04
ZFAND5	zinc finger. AN1-type domain 5	1.517	8.21E-09
EPHA2	EPH receptor A2	1.512	1.39E-06
ABTB2	ankyrin repeat and BTB (POZ) domain containing 2	1.504	3.43E-08
ADM	adrenomedullin	1.481	7.47E-08
TXNIP	thioredoxin interacting protein	1.465	1.04E-05
PMAIP1	phorbol-12-myristate-13-acetate-induced protein 1	1.426	1.10E-06
NDEL1	nudE neurodevelopment protein 1-like 1	1.422	4.95E-07
TRIB1	tribbles homolog 1 (Drosophila)	1.394	7.51E-04
CREM	cAMP responsive element modulator	1.390	1.16E-07
TXNIP	thioredoxin interacting protein	1.374	1.31E-05
PDE4B	phosphodiesterase 4B. cAMP-specific	1.373	8.64E-08
MT1M	metallothionein 1M	1.370	1.61E-06
NR1D1	nuclear receptor subfamily 1. group D. member 1	1.364	1.72E-05
BTG1	B-cell translocation gene 1. anti-proliferative	1.361	1.69E-07
BIRC3	baculoviral IAP repeat containing 3	1.349	8.81E-06
PLK2	polo-like kinase 2	1.343	1.23E-08
FEM1C	fem-1 homolog c (C. elegans)	1.334	9.72E-05
FADD	Fas (TNFRSF6)-associated via death domain	1.331	4.02E-07
MCL1	myeloid cell leukemia sequence 1 (BCL2-related)	1.330	1.77E-07
LIF	leukemia inhibitory factor	1.326	3.63E-04
NFIL3	nuclear factor. interleukin 3 regulated	1.322	6.64E-05
LPAR6	lysophosphatidic acid receptor 6	1.319	3.49E-08
IL10	interleukin 10	1.313	3.29E-06

CLDN4	claudin 4	1.309	4.82E-05
MMP12	matrix metallopeptidase 12 (macrophage elastase)	1.308	1.42E-06
TNFRSF12A	tumor necrosis factor receptor superfamily. member 12A	1.299	1.01E-04
STK17B	serine/threonine kinase 17b	1.295	1.57E-05
SOX11	SRY (sex determining region Y)-box 11	1.293	3.16E-06
OTUD1	OTU domain containing 1	1.281	1.02E-04
CDKN1A	cyclin-dependent kinase inhibitor 1A (p21. Cip1)	1.277	6.13E-04
IL1RN	interleukin 1 receptor antagonist	1.270	8.55E-08
BTG2	BTG family. member 2	1.267	7.49E-05
ZBTB10	zinc finger and BTB domain containing 10	1.264	1.54E-06
NR4A3	nuclear receptor subfamily 4. group A. member 3	1.245	2.90E-05
ETV3	ets variant 3	1.239	8.30E-05
NR0B2	nuclear receptor subfamily 0. group B. member 2	1.229	4.76E-05
C1orf116	chromosome 1 open reading frame 116	1.229	2.75E-05
TSC22D2	TSC22 domain family. member 2	1.228	1.90E-05
PLAU	plasminogen activator. urokinase	1.203	1.49E-04
EDN1	endothelin 1	1.203	4.72E-04
NFKBIE	nuclear factor of kappa light polypeptide gene enhancer in B-cells inhibitor. epsilon	1.198	2.47E-07
CD40	CD40 molecule. TNF receptor superfamily member 5	1.173	3.59E-04
THBD	thrombomodulin	1.171	3.11E-06
NR4A2	nuclear receptor subfamily 4. group A. member 2	1.165	8.92E-06
FOS	FBJ murine osteosarcoma viral oncogene homolog	1.165	6.78E-05
TBX3	T-box 3	1.156	9.31E-05
MMP13	matrix metallopeptidase 13 (collagenase 3)	1.151	2.66E-04
HIVEP2	human immunodeficiency virus type I enhancer binding protein 2	1.150	1.81E-04
VCAM1	vascular cell adhesion molecule 1	1.147	7.92E-06
BCL10	B-cell CLL/lymphoma 10	1.142	1.33E-05
EEPD1	endonuclease/exonuclease/phosphatase family domain containing 1	1.133	1.66E-05
PHLDA2	pleckstrin homology-like domain. family A. member 2	1.128	1.79E-04
RHPN2	rhophilin. Rho GTPase binding protein 2	1.127	1.51E-05
DUSP6	dual specificity phosphatase 6	1.127	5.53E-04
JMJD1C	jumonji domain containing 1C	1.121	1.36E-05
BCL6	B-cell CLL/lymphoma 6	1.119	5.51E-05
EVI2A	ecotropic viral integration site 2A	1.116	4.09E-07
XCL1	chemokine (C motif) ligand 1	1.113	1.07E-04
EFNB2	ephrin-B2	1.109	1.86E-05

RASGEF1A	RasGEF domain family. member 1A	1.103	1.04E-04
COQ10B	coenzyme Q10 homolog B (S. cerevisiae)	1.083	7.05E-05
SPRY1	sprouty homolog 1. antagonist of FGF signaling (Drosophila)	1.082	9.08E-04
TIMP1	TIMP metallopeptidase inhibitor 1	1.076	1.68E-04
TRAFD1	TRAF-type zinc finger domain containing 1	1.070	6.59E-06
CXCR4	chemokine (C-X-C motif) receptor 4	1.065	3.32E-05
BTG3	BTG family. member 3	1.063	9.92E-04
KCNK5	potassium channel. subfamily K. member 5	1.048	1.05E-04
CEBPD	CCAAT/enhancer binding protein (C/EBP). delta	1.046	8.18E-05
RHOH	ras homolog family member H	1.033	6.14E-04
REL	v-rel avian reticuloendotheliosis viral oncogene homolog	1.031	2.86E-06
TIPARP	TCDD-inducible poly(ADP-ribose) polymerase	1.027	3.48E-04
SKIL	SKI-like oncogene	1.021	2.04E-04
TNFAIP2	tumor necrosis factor. alpha-induced protein 2	1.014	4.77E-04
EGR2	early growth response 2	1.013	5.02E-05
ZFP36L1	ZFP36 ring finger protein-like 1	1.012	4.75E-06
Down-regulated genes			
CHAC1	ChaC. cation transport regulator homolog 1 (E. coli)	-1.696	9.18E-04
ABCC2	ATP-binding cassette. sub-family C (CFTR/MRP). member 2	-1.015	2.45E-06
SLC15A1	solute carrier family 15 (oligopeptide transporter). member 1	-0.851	3.56E-05
SLC9A2	solute carrier family 9. subfamily A (NHE2. cation proton antiporter 2). member 2	-0.804	9.09E-06
CCL24	chemokine (C-C motif) ligand 24	-0.784	8.75E-04
MTTP	microsomal triglyceride transfer protein	-0.755	3.26E-05
DMBT1	deleted in malignant brain tumors 1	-0.666	2.67E-04
MLEC	malectin	-0.654	9.50E-04
SSH1	slingshot protein phosphatase 1	-0.628	1.06E-03
VPS26B	vacuolar protein sorting 26 homolog B (S. pombe)	-0.610	1.04E-03
ACE2	angiotensin I converting enzyme 2	-0.607	7.35E-04
SCGB2A1	secretoglobin. family 2A. member 1	-0.594	2.74E-04
MYEOV	myeloma overexpressed	-0.592	1.58E-04
NPR3	natriuretic peptide receptor 3	-0.582	8.74E-04
CBL	Cbl proto-oncogene. E3 ubiquitin protein ligase	-0.574	3.70E-04
PLOD2	procollagen-lysine. 2-oxoglutarate 5-dioxygenase 2	-0.547	3.98E-05
C4BPA	complement component 4 binding protein. alpha	-0.525	1.04E-03
ARHGEF37	Rho guanine nucleotide exchange factor (GEF) 37	-0.521	9.19E-04

DESI2	desumoylating isopeptidase 2	-0.501	3.04E-04
STOML3	stomatin (EPB72)-like 3	-0.487	8.33E-04
UNC119B	unc-119 homolog B (C. elegans)	-0.467	2.42E-04
ZER1	zyg-11 related. cell cycle regulator	-0.455	4.92E-04
EGLN1	egl-9 family hypoxia-inducible factor 1	-0.443	2.26E-04
TCAP	titin-cap	-0.441	8.90E-04
PECAM1	platelet/endothelial cell adhesion molecule 1	-0.431	1.43E-04
ZCCHC14	zinc finger. CCHC domain containing 14	-0.430	5.16E-04
GALNT4	polypeptide N-acetylgalactosaminyltransferase 4	-0.395	6.91E-04
ANKRD13A	ankyrin repeat domain 13A	-0.388	8.85E-04
UNC45A	unc-45 homolog A (C. elegans)	-0.377	7.64E-04
TPP1	tripeptidyl peptidase I	-0.375	5.37E-04
OSBPL7	oxysterol binding protein-like 7	-0.349	1.05E-03
CPT1A	carnitine palmitoyltransferase 1A (liver)	-0.339	6.12E-04
FOXK2	forkhead box K2	-0.339	9.82E-04

Supplementary Table S3: Complete list of canonical pathways affected by DON in intestinal explants pathway

Part a: Pathways up-regulated

**Canonical Pathways**	**-log (p-value)**	**Ratio**	**Molecules**
Granulocyte Adhesion and Diapedesis	1.18E01	1.1E-01	CCL3,IL1B,MMP12,EZR,CCL20,CLDN4,CCL3L1/CCL3L3,SELE,MMP13,CXCL2,VCAM1,CXCL8, CXCR4,IL18,IL1RN,TNF,CXCR2,IL1A,CCL4,XCL1
Agranulocyte Adhesion and Diapedesis	1.13E01	1.04E-01	CCL3,IL1B,MMP12,EZR,CCL20,CLDN4,CCL3L1/CCL3L3,SELE,MMP13,CXCL2,VCAM1,CXCL8, CXCR4,IL18,IL1RN,TNF,CXCR2,IL1A,CCL4,XCL1
Glucocorticoid Receptor Signaling	1.11E01	7.69E-02	HSPA2,NFKB1,CCL3,CSF2,JAK2,IL1B,PLAU,SELE,NFKBIE,NFATC1,NFKBIA,VCAM1,CXCL8, FOS,IL1RN,DUSP1,SGK1,NR3C1,TNF,SMAD3,CDKN1A,IL10,FOXO3
Differential Regulation of Cytokine Production in Intestinal Epithelial Cells by IL-17A and IL-17F	1.07E01	3.91E-01	CCL3,CSF2,IL1B,TNF,IL1A,IL17A,IL10,CCL4,IL17F
Communication between Innate and Adaptive Immune Cells	1.01E01	1.25E-01	CCR7,CCL3,CSF2,IL1B,CCL3L1/CCL3L3,CD40,CXCL8,CD83,IL18,IL1RN,TNF,IL1A,IL10,CCL4
Differential Regulation of Cytokine Production in Macrophages and T Helper Cells by IL-17A and IL-17F	1.01E01	4.44E-01	CCL3,CSF2,IL1B,TNF,IL17A,IL10,CCL4,IL17F
Hepatic Fibrosis / Hepatic Stellate Cell Activation	9.55E00	1.03E-01	CCR7,NFKB1,IL1B,EDNRB,MMP13,CD40,VCAM1,CXCL8,IFNGR1,TNF,SMAD3,IL1A,EDN1,IL4R, IL10,TIMP1
IL-10 Signaling	9.48E00	1.54E-01	FOS,IL18,NFKB1,IL1RN,IL1B,SOCS3,TNF,IL1A,IL4R,IL10,NFKBIE,NFKBIA
T Helper Cell Differentiation	9.41E00	1.67E-01	GATA3,IFNGR1,BCL6,IL18,STAT4,TNF,ICOSLG/LOC102723996,IL17A,IL4R,IL10,CD40,IL17F
Atherosclerosis Signaling	9.4E00	1.08E-01	NFKB1,IL1B,SELE,MMP13,CD40,VCAM1,CXCL8,CXCR4,IL18,IL1RN,TNF,IL1A,F3,TNFRSF12A, PLA2G4A
Role of Cytokines in Mediating Communication between Immune Cells	9.36E00	2E-01	IL18,IL1RN,CSF2,IL1B,TNF,IL22,IL1A,IL17A,IL10,IL17F,CXCL8
TREM1 Signaling	9.01E00	1.47E-01	CD83,IL18,NFKB1,CCL3,CSF2,IL1B,JAK2,TNF,IL10,CD40,CXCL8
Role of Macrophages, Fibroblasts and Endothelial Cells in Rheumatoid Arthritis	8.92E00	6.43E-02	NFKB1,CEBPD,CSF2,JAK2,IL1B,SOCS3,SELE,NFKBIE,MMP13,NFATC1,NFKBIA,VCAM1, CXCL8,FOS,IL18,IL1RN,CREB5,TNF,IL1A,IL17A,IL10,IRAK2
TNFR1 Signaling	8.83E00	1.85E-01	FOS,BIRC2,NFKB1,CYCS,TNFAIP3,TNF,FADD,NFKBIE,NFKBIA,BIRC3
TNFR2 Signaling	8.28E00	2.35E-01	FOS,BIRC2,NFKB1,TNFAIP3,TNF,NFKBIE,NFKBIA,BIRC3

Dendritic Cell Maturation	7.97E00	7.58E-02	CCR7,NFKB1,STAT4,CSF2,JAK2,IL1B,NFKBIE,CD40,NFKBIA,CD83,IL18,IL1RN,CREB5,TNF,IL1A,IL10
HMGB1 Signaling	7.87E00	1.1E-01	FOS,IFNGR1,RHOH,NFKB1,TNF,RHOB,IL1A,PLAT,SELE,RND3,VCAM1,CXCL8
Role of Hypercytokinemia/hyperchemokinemia in the Pathogenesis of Influenza	7.84E00	1.96E-01	IL18,CCL3,IL1RN,IL1B,TNF,IL1A,IL17A,CCL4,CXCL8
PPAR Signaling	7.82E00	1.12E-01	FOS,IL18,NFKB1,IL1RN,IL1B,TNF,IL1A,NFKBIE,PPARG,NFKBIA,CITED2,NR0B2
IL-6 Signaling	7.72E00	1.05E-01	NFKB1,JAK2,IL1B,MCL1,SOCS3,NFKBIE,NFKBIA,CXCL8,FOS,IL18,IL1RN,TNF,IL1A
TWEAK Signaling	7.66E00	2.05E-01	BIRC2,NFKB1,CYCS,TNFRSF12A,FADD,NFKBIE,NFKBIA,BIRC3
Role of Osteoblasts, Osteoclasts and Chondrocytes in Rheumatoid Arthritis	7.45E00	6.8E-02	BIRC2,NFKB1,CSF2,IL1B,NFKBIE,BMP2,MMP13,NFATC1,NFKBIA,BIRC3,FOS,IL18,IL1RN,TNF,IL1A,IL17A,IL10
IL-17A Signaling in Fibroblasts	7.44E00	2E-01	FOS,NFKB1,CEBPD,IL17A,NFKBIE,NFKBIZ,NFKBIA,NFKBID
Altered T Cell and B Cell Signaling in Rheumatoid Arthritis	7.22E00	1.1E-01	IL18,NFKB1,IL1RN,CSF2,IL1B,TNF,IL22,IL1A,IL17A,IL10,CD40
p38 MAPK Signaling	6.7E00	1E-01	DUSP10,IL18,IL1RN,DUSP1,IL1B,CREB5,TNF,IL1A,H3F3A/H3F3B,FADD,IRAK2,PLA2G4A
Induction of Apoptosis by HIV1	6.68E00	1.34E-01	BIRC2,CXCR4,NFKB1,CYCS,TNF,FADD,NFKBIE,NFKBIA,BIRC3
iNOS Signaling	6.61E00	1.51E-01	FOS,IRF1,IFNGR1,NFKB1,JAK2,NFKBIE,IRAK2,NFKBIA
Role of IL-17F in Allergic Inflammatory Airway Diseases	6.53E00	1.67E-01	NFKB1,CSF2,IL1B,CREB5,CCL4,MMP13,IL17F,CXCL8
NF-κB Signaling	6.48E00	7.73E-02	NFKB1,IL1B,BMP2,NFKBIE,FADD,CD40,NFKBIA,PELI1,IL18,IL1RN,TNFAIP3,BCL10,TNF,IL1A
Type I Diabetes Mellitus Signaling	6.2E00	9.09E-02	IRF1,IFNGR1,NFKB1,CYCS,IL1B,JAK2,SOCS3,TNF,FADD,NFKBIE,NFKBIA
IL-17A Signaling in Gastric Cells	5.84E00	2.14E-01	FOS,NFKB1,TNF,CCL20,IL17A,CXCL8
Acute Phase Response Signaling	5.81E00	7.18E-02	NFKB1,JAK2,IL1B,SOCS3,NFKBIE,NFKBIA,FOS,IL18,IL1RN,NR3C1,TNF,IL1A,HAMP
Role of PKR in Interferon Induction and Antiviral Response	5.73E00	1.43E-01	IRF1,NFKB1,CYCS,TNF,FADD,NFKBIE,NFKBIA
Death Receptor Signaling	5.66E00	1.18E-01	BIRC2,NFKB1,CYCS,TNF,FADD,NFKBIE,NFKBIA,BIRC3
Cholecystokinin/Gastrin-mediated Signaling	5.57E00	9.43E-02	FOS,RHOH,IL18,CREM,IL1RN,IL1B,TNF,RHOB,IL1A,RND3
Activation of IRF by Cytosolic Pattern Recognition Receptors	5.38E00	1.08E-01	NFKB1,TNF,IFIH1,IL10,FADD,CD40,NFKBIE,NFKBIA
IL-12 Signaling and Production in Macrophages	5.27E00	7.01E-02	FOS,REL,IRF1,IFNGR1,IL18,NFKB1,STAT4,TNF,IL10,CD40,PPARG
Apoptosis Signaling	5.19E00	9E-02	BIRC2,NFKB1,CYCS,MCL1,BCL2A1,TNF,NFKBIE,NFKBIA,BIRC3
Hepatic Cholestasis	5.12E00	6.01E-02	IL18,NFKB1,IL1RN,IL1B,TNF,IL1A,NFKBIE,IRAK2,NFKBIA,CXCL8,NR0B2
Role of IL-17A in Arthritis	4.83E00	1.09E-01	NFKB1,CCL20,IL17A,NFKBIE,MMP13,NFKBIA,CXCL8
PI3K Signaling in B Lymphocytes	4.65E00	6.99E-02	FOS,NFKB1,BCL10,IL4R,CD40,NFKBIE,NFKBIA,NFATC1,FOXO3,ATF3
Glioma Invasiveness Signaling	4.62E00	1.06E-01	RHOH,ITGAV,PLAU,RHOB,PLAUR,TIMP1,RND3
IL-8 Signaling	4.6E00	5.33E-02	FOS,HBEGF,RHOH,NFKB1,ANGPT2,ITGAV,RHOB,CXCR2,IRAK2,RND3,VCAM1,CXCL8

Role of Tissue Factor in Cancer	4.37E00	6.92E-02	HBEGF,CSF2,ITGAV,IL1B,JAK2,PLAUR,F3,MMP13,CXCL8
ERK5 Signaling	4.34E00	1.03E-01	FOS,CREB5,SGK1,LIF,FOXO3,FOSL1,SH2D2A
Molecular Mechanisms of Cancer	4.23E00	4.12E-02	BIRC2,RHOH,NFKB1,JAK2,BMP2,NFKBIE,FADD,NFKBIA,BIRC3,FOS,CYCS,SMAD3,RHOB, CDKN1A,RND3,PMAIP1
Crosstalk between Dendritic Cells and Natural Killer Cells	4.23E00	7.55E-02	CCR7,CD83,IL18,NFKB1,CSF2,TNF,CD40,CD69
Production of Nitric Oxide and Reactive Oxygen Species in Macrophages	4.1E00	5.19E-02	FOS,IRF1,IFNGR1,RHOH,NFKB1,JAK2,TNF,RHOB,NFKBIE,NFKBIA,RND3
MIF-mediated Glucocorticoid Regulation	3.92E00	1.19E-01	NFKB1,NR3C1,NFKBIE,NFKBIA,PLA2G4A
ERK/MAPK Signaling	3.91E00	5.21E-02	FOS,DUSP1,CREB5,H3F3A/H3F3B,DUSP6,ETS1,PPARG,ELF3,NFATC1,PLA2G4A,ETS2
Lymphotoxin β Receptor Signaling	3.83E00	9.68E-02	BIRC2,NFKB1,CYCS,NFKBIA,NFKBID,VCAM1
Coagulation System	3.8E00	1.32E-01	PLAU,THBD,PLAUR,F3,PLAT
Regulation of IL-2 Expression in Activated and Anergic T Lymphocytes	3.76E00	7.87E-02	FOS,NFKB1,BCL10,SMAD3,NFKBIE,NFKBIA,NFATC1
GADD45 Signaling	3.71E00	1.67E-01	CDKN1A,GADD45B,GADD45A,GADD45G
April Mediated Signaling	3.62E00	1.14E-01	FOS,NFKB1,NFKBIE,NFKBIA,NFATC1
ATM Signaling	3.58E00	9.09E-02	CREB5,CDKN1A,GADD45B,GADD45A,NFKBIA,GADD45G
FXR/RXR Activation	3.53E00	6.36E-02	IL18,IL1RN,IL1B,TNF,IL1A,PPARG,NR0B2
B Cell Activating Factor Signaling	3.52E00	1.09E-01	FOS,NFKB1,NFKBIE,NFKBIA,NFATC1
RANK Signaling in Osteoclasts	3.49E00	7.22E-02	FOS,BIRC2,NFKB1,NFKBIE,NFKBIA,NFATC1,BIRC3
MIF Regulation of Innate Immunity	3.47E00	9.62E-02	FOS,NFKB1,NFKBIE,NFKBIA,PLA2G4A
CD40 Signaling	3.46E00	8.45E-02	FOS,NFKB1,TNFAIP3,CD40,NFKBIE,NFKBIA
IL-17A Signaling in Airway Cells	3.42E00	7.89E-02	NFKB1,JAK2,CCL20,IL17A,NFKBIE,NFKBIA
Role of RIG1-like Receptors in Antiviral Innate Immunity	3.32E00	1.02E-01	NFKB1,IFIH1,FADD,NFKBIE,NFKBIA
Erythropoietin Signaling	3.32E00	7.59E-02	FOS,NFKB1,JAK2,SOCS3,NFKBIE,NFKBIA
PI3K/AKT Signaling	3.29E00	5.26E-02	NFKB1,JAK2,MCL1,CDKN1A,NOS3,NFKBIE,NFKBIA,FOXO3
Tec Kinase Signaling	3.25E00	4.89E-02	FOS,RHOH,NFKB1,STAT4,JAK2,TNF,RHOB,FADD,RND3
Graft-versus-Host Disease Signaling	3.23E00	9.8E-02	IL18,IL1RN,IL1B,TNF,IL1A
Antioxidant Action of Vitamin C	3.21E00	6.31E-02	NFKB1,CSF2,JAK2,TNF,NFKBIE,NFKBIA,PLA2G4A
B Cell Receptor Signaling	3.19E00	5.14E-02	BCL6,NFKB1,BCL10,CREB5,BCL2A1,ETS1,NFKBIE,NFKBIA,NFATC1
IL-17 Signaling	3.15E00	8E-02	NFKB1,JAK2,IL17A,TIMP1,IL17F,CXCL8
Role of IL-17A in Psoriasis	3.08E00	2.14E-01	CCL20,IL17A,CXCL8
CD27 Signaling in Lymphocytes	3.02E00	8.47E-02	FOS,NFKB1,CYCS,NFKBIE,NFKBIA
Hematopoiesis from Pluripotent Stem Cells	3.02E00	7.94E-02	CSF2,IL1A,LIF,IL10,CXCL8
Colorectal Cancer Metastasis Signaling	2.98E00	4.1E-02	FOS,IFNGR1,RHOH,NFKB1,JAK2,TNF,RHOB,SMAD3,MMP12,MMP13,RND3

VDR/RXR Activation	2.96E00	6.82E-02	CSF2,THBD,CDKN1A,MXD1,GADD45A,KLF4
4-1BB Signaling in T Lymphocytes	2.96E00	1.11E-01	NFKB1,TNFRSF9,NFKBIE,NFKBIA
Toll-like Receptor Signaling	2.84E00	7.81E-02	FOS,NFKB1,TNFAIP3,IRAK2,NFKBIA
Gαq Signaling	2.76E00	4.68E-02	RHOH,NFKB1,RHOB,RGS2,NFKBIE,NFKBIA,NFATC1,RND3
Type II Diabetes Mellitus Signaling	2.75E00	4.09E-02	ACSL4,NFKB1,SOCS3,TNF,NFKBIE,PPARG,NFKBIA
Protein Kinase A Signaling	2.73E00	3.42E-02	DUSP10,NFKB1,H3F3A/H3F3B,NFKBIE,PDE4B,H1F0,NFATC1,NFKBIA,CREM,CREB5,DUSP1, SMAD3,DUSP6,NOS3
Systemic Lupus Erythematosus Signaling	2.71E00	3.91E-02	FOS,IL18,CREM,IL1RN,IL1B,TNF,IL1A,IL10,CD40,NFATC1
ILK Signaling	2.7E00	4.39E-02	FOS,RHOH,NFKB1,CREB5,SNAI2,TNF,RHOB,BMP2,RND3
IL-1 Signaling	2.57E00	5.5E-02	FOS,NFKB1,IL1A,NFKBIE,IRAK2,NFKBIA
Angiopoietin Signaling	2.55E00	6.67E-02	NFKB1,ANGPT2,NOS3,NFKBIE,NFKBIA
Hypoxia Signaling in the Cardiovascular System	2.52E00	7.35E-02	CREB5,EDN1,NOS3,NFKBIE,NFKBIA
JAK/Stat Signaling	2.49E00	7.04E-02	FOS,STAT4,JAK2,SOCS3,CDKN1A
Neurotrophin/TRK Signaling	2.46E00	6.58E-02	FOS,CREB5,SPRY2,SPRY1,BDNF
p53 Signaling	2.46E00	5.31E-02	SNAI2,CDKN1A,GADD45B,GADD45A,PMAIP1,GADD45G
Small Cell Lung Cancer Signaling	2.38E00	5.32E-02	BIRC2,NFKB1,CYCS,NFKBIE,NFKBIA
PEDF Signaling	2.38E00	6.33E-02	NFKB1,NFKBIE,PPARG,NFKBIA,BDNF
Prolactin Signaling	2.33E00	5.95E-02	FOS,IRF1,JAK2,SOCS3,NR3C1
Aryl Hydrocarbon Receptor Signaling	2.3E00	4.09E-02	FOS,NFKB1,IL1B,TNF,IL1A,CDKN1A,NR0B2
RAR Activation	2.29E00	4.1E-02	FOS,REL,NFKB1,DUSP1,JAK2,SMAD3,BMP2,CITED2
Role of JAK1, JAK2 and TYK2 in Interferon Signaling	2.28E00	1.07E-01	IFNGR1,NFKB1,JAK2
iCOS-iCOSL Signaling in T Helper Cells	2.27E00	4.76E-02	NFKB1,ICOSLG/LOC102723996,CD40,NFKBIE,NFKBIA,NFATC1
G-Protein Coupled Receptor Signaling	2.26E00	3.62E-02	NFKB1,DUSP1,CREB5,CXCR2,RGS2,ADORA3,DUSP6,NFKBIE,PDE4B,NFKBIA
Airway Pathology in Chronic Obstructive Pulmonary Disease	2.23E00	1.82E-01	TNF,CXCL8
IL-15 Production	2.13E00	9.68E-02	IRF1,NFKB1,JAK2
Pathogenesis of Multiple Sclerosis	2.13E00	2E-01	CCL3,CCL4
Semaphorin Signaling in Neurons	2.13E00	7.41E-02	RHOH,RHOB,RND3,RND1
CD28 Signaling in T Helper Cells	2.1E00	4.41E-02	FOS,NFKB1,BCL10,NFKBIE,NFKBIA,NFATC1
PKCθ Signaling in T Lymphocytes	2.1E00	4.17E-02	FOS,NFKB1,BCL10,NFKBIE,NFKBIA,NFATC1
Prostate Cancer Signaling	2.1E00	4.85E-02	NFKB1,CREB5,CDKN1A,NFKBIE,NFKBIA
LXR/RXR Activation	2.02E00	4.32E-02	IL18,NFKB1,IL1RN,IL1B,TNF,IL1A
TGF-β Signaling	2.01E00	5.32E-02	FOS,INHBA,SMAD3,PMEPA1,BMP2
Leukocyte Extravasation Signaling	2.01E00	3.81E-02	RHOH,CXCR4,MMP12,EZR,CLDN4,TIMP1,MMP13,VCAM1

IL-15 Signaling	1.89E00	5.56E-02	NFKB1,CSF2,JAK2,IL17A
SAPK/JNK Signaling	1.86E00	4.76E-02	DUSP10,GADD45A,FADD,NFATC1,SH2D2A
Circadian Rhythm Signaling	1.86E00	7.89E-02	BHLHE40,CREB5,NR1D1
IL-9 Signaling	1.86E00	7.5E-02	NFKB1,SOCS3,TNF
Oncostatin M Signaling	1.86E00	8.57E-02	JAK2,PLAU,MMP13
Interferon Signaling	1.86E00	8.33E-02	IRF1,IFNGR1,JAK2
Role of Pattern Recognition Receptors in Recognition of Bacteria and Viruses	1.84E00	4.59E-02	NFKB1,IL1B,TNF,IFIH1,IL10
GM-CSF Signaling	1.84E00	5.88E-02	CSF2,JAK2,BCL2A1,ETS1
T Cell Receptor Signaling	1.82E00	4.59E-02	FOS,NFKB1,BCL10,NFKBIA,NFATC1
Relaxin Signaling	1.77E00	3.66E-02	FOS,NFKB1,NOS3,NFKBIE,PDE4B,NFKBIA
HGF Signaling	1.77E00	4.5E-02	FOS,CDKN1A,ETS1,ELF3,ETS2
PPARα/RXRα Activation	1.77E00	3.5E-02	NFKB1,IL1B,JAK2,SMAD3,NFKBIE,NFKBIA,NR0B2
cAMP-mediated signaling	1.75E00	3.54E-02	CREM,DUSP1,CREB5,CXCR2,RGS2,ADORA3,DUSP6,PDE4B
PXR/RXR Activation	1.75E00	4.35E-02	NR3C1,TNF,FOXO3,NR0B2
Retinoic acid Mediated Apoptosis Signaling	1.75E00	5.48E-02	TIPARP,IRF1,CYCS,FADD
LPS/IL-1 Mediated Inhibition of RXR Function	1.74E00	3.27E-02	ACSL4,IL18,IL1RN,IL1B,TNF,IL1A,SMOX,NR0B2
Docosahexaenoic Acid (DHA) Signaling	1.7E00	6E-02	CYCS,IL1B,BCL2A1
Inhibition of Matrix Metalloproteases	1.7E00	7.5E-02	MMP12,TIMP1,MMP13
Pancreatic Adenocarcinoma Signaling	1.66E00	3.91E-02	HBEGF,NFKB1,JAK2,SMAD3,CDKN1A
Extrinsic Prothrombin Activation Pathway	1.64E00	9.09E-02	THBD,F3
LPS-stimulated MAPK Signaling	1.63E00	4.82E-02	FOS,NFKB1,NFKBIE,NFKBIA
NF-κB Activation by Viruses	1.61E00	4.82E-02	NFKB1,ITGAV,NFKBIE,NFKBIA
IL-4 Signaling	1.61E00	5E-02	JAK2,NR3C1,IL4R,NFATC1
Corticotropin Releasing Hormone Signaling	1.57E00	3.45E-02	FOS,JUND,CREB5,NOS3,BDNF
Integrin Signaling	1.54E00	3.37E-02	RHOH,ITGAV,RHOB,NEDD9,BCAR3,ARF6,RND3
Thiosulfate Disproportionation III (Rhodanese)	1.53E00	1.67E-01	MOCS3
Ceramide Signaling	1.5E00	4.4E-02	FOS,NFKB1,CYCS,TNF
Polyamine Regulation in Colon Cancer	1.38E00	6.67E-02	MXD1,PPARG
Role of NFAT in Regulation of the Immune Response	1.36E00	3E-02	FOS,NFKB1,RCAN1,NFKBIE,NFKBIA,NFATC1
Bladder Cancer Signaling	1.36E00	4.12E-02	MMP12,CDKN1A,MMP13,CXCL8
IL-22 Signaling	1.31E00	8E-02	SOCS3,IL22
Tumoricidal Function of Hepatic Natural Killer Cells	1.31E00	7.41E-02	CYCS,FADD
Actin Nucleation by ARP-WASP Complex	1.29E00	4.48E-02	RHOH,RHOB,RND3

Role of JAK family kinases in IL-6-type Cytokine Signaling	1.28E00	7.14E-02	JAK2,SOCS3
Regulation of the Epithelial-Mesenchymal Transition Pathway	1.27E00	3.06E-02	NFKB1,JAK2,ID2,SNAI2,SMAD3,ETS1
IGF-1 Signaling	1.24E00	3.74E-02	FOS,JAK2,SOCS3,FOXO3
Airway Inflammation in Asthma	1.23E00	1.67E-01	TNF
Spermine and Spermidine Degradation I	1.23E00	7.14E-02	SMOX
Melatonin Degradation II	1.23E00	8.33E-02	SMOX
Molybdenum Cofactor Biosynthesis	1.23E00	6.25E-02	MOCS3
Phospholipase C Signaling	1.21E00	2.64E-02	RHOH,NFKB1,CREB5,RHOB,NFATC1,PLA2G4A,RND3
Telomerase Signaling	1.19E00	3.77E-02	CDKN1A,ETS1,ELF3,ETS2
Role of JAK1 and JAK3 in γc Cytokine Signaling	1.19E00	4.41E-02	JAK2,SOCS3,IL4R
Estrogen-Dependent Breast Cancer Signaling	1.17E00	4.11E-02	FOS,NFKB1,CREB5
HIF1α Signaling	1.17E00	3.57E-02	MMP12,EDN1,NOS3,MMP13
Tetrahydrofolate Salvage from 5,10-methenyltetrahydrofolate	1.14E00	1E-01	MTHFD1L
Citrulline-Nitric Oxide Cycle	1.14E00	6.25E-02	NOS3
Role of PI3K/AKT Signaling in the Pathogenesis of Influenza	1.14E00	3.95E-02	NFKB1,NFKBIE,NFKBIA
Tight Junction Signaling	1.1E00	2.99E-02	FOS,NFKB1,MPP5,TNF,CLDN4
CXCR4 Signaling	1.1E00	2.87E-02	FOS,RHOH,CXCR4,RHOB,RND3
fMLP Signaling in Neutrophils	1.1E00	3.03E-02	NFKB1,NFKBIE,NFKBIA,NFATC1
Renin-Angiotensin Signaling	1.1E00	3.17E-02	FOS,NFKB1,JAK2,TNF
Growth Hormone Signaling	1.08E00	3.85E-02	FOS,JAK2,SOCS3
Chemokine Signaling	1.08E00	4E-02	FOS,CXCR4,CCL4
CCR5 Signaling in Macrophages	1.06E00	3.09E-02	FOS,CCL3,CCL4
Role of JAK2 in Hormone-like Cytokine Signaling	1.04E00	5.41E-02	JAK2,SOCS3
STAT3 Pathway	1.02E00	3.75E-02	JAK2,SOCS3,CDKN1A
Hereditary Breast Cancer Signaling	1.02E00	2.99E-02	CDKN1A,GADD45B,GADD45A,GADD45G
Cell Cycle Regulation by BTG Family Proteins	1.02E00	5.13E-02	BTG1,BTG2
Folate Polyglutamylation	1E00	5E-02	MTHFD1L
Gα12/13 Signaling	1E00	3.15E-02	LPAR6,NFKB1,NFKBIE,NFKBIA
RhoA Signaling	1E00	3.25E-02	LPAR6,EZR,RHPN2,RND3
VEGF Family Ligand-Receptor Interactions	9.81E-01	3.41E-02	FOS,NOS3,PLA2G4A
BMP signaling pathway	9.81E-01	3.49E-02	NFKB1,FST,BMP2
Histidine Degradation III	9.46E-01	5.56E-02	MTHFD1L

Tryptophan Degradation to 2-amino-3-carboxymuconate Semialdehyde	9.46E-01	5.56E-02	IDO1
Endothelin-1 Signaling	9.39E-01	2.6E-02	FOS,EDN1,EDNRB,NOS3,PLA2G4A
Thyroid Cancer Signaling	9.23E-01	4.55E-02	PPARG,BDNF
Ephrin Receptor Signaling	9.16E-01	2.38E-02	EFNB2,CXCR4,JAK2,CREB5,EPHA2
Regulation of Actin-based Motility by Rho	8.96E-01	3.26E-02	RHOH,RHOB,RND3
Allograft Rejection Signaling	8.96E-01	3.09E-02	TNF,IL10,CD40
Signaling by Rho Family GTPases	8.79E-01	2.28E-02	FOS,RHOH,NFKB1,RHOB,EZR,RND3
ErbB Signaling	8.74E-01	3.33E-02	FOS,HBEGF,AREG
Insulin Receptor Signaling	8.7E-01	2.68E-02	JAK2,SOCS3,SGK1,FOXO3
OX40 Signaling Pathway	8.63E-01	3.09E-02	NFKB1,NFKBIE,NFKBIA
Folate Transformations I	8.55E-01	3.03E-02	MTHFD1L
Cell Cycle: G2/M DNA Damage Checkpoint Regulation	8.55E-01	4.08E-02	CDKN1A,GADD45A
Human Embryonic Stem Cell Pluripotency	8.44E-01	2.47E-02	INHBA,SMAD3,BMP2,BDNF
MSP-RON Signaling Pathway	8.39E-01	3.92E-02	JAK2,TNF
UVA-Induced MAPK Signaling	8.11E-01	3.06E-02	FOS,TIPARP,CYCS
Autoimmune Thyroid Disease Signaling	8.09E-01	3.23E-02	IL10,CD40
Thrombin Signaling	8.03E-01	2.37E-02	GATA3,RHOH,NFKB1,RHOB,RND3
VEGF Signaling	8.01E-01	2.75E-02	NOS3,FOXO3,SH2D2A
Fcγ Receptor-mediated Phagocytosis in Macrophages and Monocytes	7.91E-01	2.83E-02	CSF2,EZR,ARF6
Hematopoiesis from Multipotent Stem Cells	7.83E-01	8.33E-02	CSF2
Chronic Myeloid Leukemia Signaling	7.82E-01	2.83E-02	NFKB1,SMAD3,CDKN1A
Mouse Embryonic Stem Cell Pluripotency	7.72E-01	3.03E-02	JAK2,ID2,LIF
Glioblastoma Multiforme Signaling	7.52E-01	2.38E-02	RHOH,RHOB,CDKN1A,RND3
Fatty Acid Activation	7.51E-01	5.26E-02	ACSL4
Amyotrophic Lateral Sclerosis Signaling	7.36E-01	2.38E-02	BIRC2,CYCS,BIRC3
UVB-Induced MAPK Signaling	7.28E-01	3.45E-02	FOS,H3F3A/H3F3B
Superpathway of Citrulline Metabolism	7.22E-01	2.63E-02	NOS3
Phenylalanine Degradation IV (Mammalian, via Side Chain)	7.22E-01	2.56E-02	SMOX
Thrombopoietin Signaling	7.04E-01	3.12E-02	FOS,JAK2
Nur77 Signaling in T Lymphocytes	7.04E-01	3.12E-02	CYCS,NFATC1
Axonal Guidance Signaling	7E-01	1.85E-02	EFNB2,CXCR4,BMP2,MMP13,EPHA2,NFATC1,BDNF,RND1,ADAMTS1

NAD biosynthesis II (from tryptophan)	6.95E-01	2.86E-02	IDO1
Germ Cell-Sertoli Cell Junction Signaling	6.9E-01	2.37E-02	RHOH,TNF,RHOB,RND3
Granzyme B Signaling	6.7E-01	5.56E-02	CYCS
Parkinson's Signaling	6.7E-01	5.26E-02	CYCS
Myc Mediated Apoptosis Signaling	6.69E-01	3.17E-02	CYCS,FADD
Xenobiotic Metabolism Signaling	6.6E-01	2.08E-02	NFKB1,IL1B,TNF,IL1A,SMOX,CITED2
Fc Epsilon RI Signaling	6.54E-01	2.56E-02	CSF2,TNF,PLA2G4A
Role of BRCA1 in DNA Damage Response	6.48E-01	2.82E-02	CDKN1A,GADD45A
γ-linolenate Biosynthesis II (Animals)	6.47E-01	4.17E-02	ACSL4
Mitochondrial L-carnitine Shuttle Pathway	6.47E-01	4.55E-02	ACSL4
Putrescine Degradation III	6.47E-01	3.33E-02	SMOX
Sphingosine-1-phosphate Signaling	6.46E-01	2.44E-02	RHOH,RHOB,RND3
Role of NANOG in Mammalian Embryonic Stem Cell Pluripotency	6.31E-01	2.52E-02	JAK2,LIF,BMP2
Tryptophan Degradation X (Mammalian, via Tryptamine)	6.25E-01	3.45E-02	SMOX
Huntington's Disease Signaling	6.2E-01	1.98E-02	HSPA2,CYCS,CREB5,SGK1,BDNF
Cardiomyocyte Differentiation via BMP Receptors	6.05E-01	4.55E-02	BMP2
Granzyme A Signaling	6.05E-01	5E-02	H1F0
1D-myo-inositol Hexakisphosphate Biosynthesis II (Mammalian)	6.05E-01	3.57E-02	ITPKC
D-myo-inositol (1,3,4)-trisphosphate Biosynthesis	6.05E-01	4E-02	ITPKC
PTEN Signaling	5.96E-01	2.17E-02	NFKB1,CDKN1A,FOXO3
Cell Cycle: G1/S Checkpoint Regulation	5.88E-01	2.78E-02	SMAD3,CDKN1A
DNA Methylation and Transcriptional Repression Signaling	5.86E-01	4.35E-02	ARID4B
RhoGDI Signaling	5.82E-01	1.98E-02	RHOH,RHOB,EZR,RND3
Role of MAPK Signaling in the Pathogenesis of Influenza	5.7E-01	2.78E-02	TNF,PLA2G4A
Tryptophan Degradation III (Eukaryotic)	5.68E-01	2.08E-02	IDO1
P2Y Purigenic Receptor Signaling Pathway	5.63E-01	2.08E-02	FOS,NFKB1,CREB5
D-myo-inositol (1,4,5,6)-Tetrakisphosphate Biosynthesis	5.56E-01	2.08E-02	DUSP10,DUSP1,SOCS3
D-myo-inositol (3,4,5,6)-tetrakisphosphate Biosynthesis	5.56E-01	2.08E-02	DUSP10,DUSP1,SOCS3
Renal Cell Carcinoma Signaling	5.52E-01	2.53E-02	FOS,ETS1

NRF2-mediated Oxidative Stress Response	5.51E-01	2.05E-02	FOS,JUND,FOSL1,ENC1
IL-3 Signaling	5.44E-01	2.67E-02	FOS,JAK2
Estrogen Receptor Signaling	5.32E-01	2.21E-02	NR3C1,H3F3A/H3F3B,NR0B2
Ephrin B Signaling	5.19E-01	2.44E-02	EFNB2,CXCR4
Estrogen-mediated S-phase Entry	5.19E-01	3.57E-02	CDKN1A
Dopamine Degradation	5.19E-01	2.63E-02	SMOX
Superpathway of D-myo-inositol (1,4,5)-trisphosphate Metabolism	5.19E-01	3.03E-02	ITPKC
GNRH Signaling	5.14E-01	1.96E-02	FOS,NFKB1,CREB5
FLT3 Signaling in Hematopoietic Progenitor Cells	5.12E-01	2.53E-02	STAT4,CREB5
Leptin Signaling in Obesity	5.12E-01	2.35E-02	JAK2,SOCS3
eNOS Signaling	5.08E-01	1.94E-02	HSPA2,LPAR6,NOS3
HER-2 Signaling in Breast Cancer	5.04E-01	2.44E-02	AREG,CDKN1A
Superpathway of Inositol Phosphate Compounds	5.02E-01	1.71E-02	DUSP10,DUSP1,SOCS3,ITPKC
PDGF Signaling	4.96E-01	2.33E-02	FOS,JAK2
Lipid Antigen Presentation by CD1	4.9E-01	3.33E-02	ARF6
Antiproliferative Role of TOB in T Cell Signaling	4.9E-01	3.85E-02	SMAD3
Cytotoxic T Lymphocyte-mediated Apoptosis of Target Cells	4.68E-01	2.27E-02	CYCS,FADD
Intrinsic Prothrombin Activation Pathway	4.64E-01	2.7E-02	THBD
Pyrimidine Ribonucleotides Interconversion	4.64E-01	2.5E-02	ENTPD7
D-myo-inositol-5-phosphate Metabolism	4.6E-01	1.85E-02	DUSP10,DUSP1,SOCS3
3-phosphoinositide Degradation	4.6E-01	1.85E-02	DUSP10,DUSP1,SOCS3
Pyrimidine Ribonucleotides De Novo Biosynthesis	4.4E-01	1.85E-02	ENTPD7
Fatty Acid β-oxidation I	4.4E-01	2.22E-02	ACSL4
Neuregulin Signaling	4.22E-01	1.92E-02	HBEGF,AREG
B Cell Development	4.18E-01	2.78E-02	CD40
Retinoate Biosynthesis I	4.18E-01	2.7E-02	BMP2
Serotonin Receptor Signaling	4.08E-01	2.04E-02	SMOX
3-phosphoinositide Biosynthesis	4.03E-01	1.66E-02	DUSP10,DUSP1,SOCS3
Aldosterone Signaling in Epithelial Cells	3.98E-01	1.78E-02	HSPA2,DUSP1,SGK1
G Protein Signaling Mediated by Tubby	3.98E-01	2.27E-02	JAK2
Inhibition of Angiogenesis by TSP1	3.98E-01	2.38E-02	NOS3
tRNA Splicing	3.88E-01	2.17E-02	PDE4B
Stearate Biosynthesis I (Animals)	3.88E-01	2.04E-02	ACSL4

Noradrenaline and Adrenaline Degradation	3.79E-01	1.89E-02	SMOX
Neuropathic Pain Signaling In Dorsal Horn Neurons	3.56E-01	1.83E-02	FOS,BDNF
Netrin Signaling	3.53E-01	1.72E-02	NFATC1
Paxillin Signaling	3.51E-01	1.71E-02	ITGAV,ARF6
Transcriptional Regulatory Network in Embryonic Stem Cells	3.45E-01	2.33E-02	SKIL
Melanoma Signaling	3.3E-01	2E-02	CDKN1A
UVC-Induced MAPK Signaling	3.3E-01	2.27E-02	FOS
NGF Signaling	3.23E-01	1.64E-02	NFKB1,CREB5
Role of Oct4 in Mammalian Embryonic Stem Cell Pluripotency	3.08E-01	1.92E-02	JARID2
Sertoli Cell-Sertoli Cell Junction Signaling	3.07E-01	1.52E-02	TNF,CLDN4,NOS3
Androgen Signaling	3.02E-01	1.38E-02	NFKB1,SMAD3
Gαs Signaling	3.02E-01	1.6E-02	CREB5,RGS2
Calcium Signaling	2.97E-01	1.38E-02	CREB5,RCAN1,NFATC1
Ephrin A Signaling	2.89E-01	1.85E-02	EPHA2
14-3-3-mediated Signaling	2.82E-01	1.65E-02	FOS,TNF
Protein Ubiquitination Pathway	2.76E-01	1.48E-02	HSPA2,BIRC2,NEDD4L,BIRC3
Sperm Motility	2.74E-01	1.39E-02	PDE4B,PLA2G4A
CNTF Signaling	2.66E-01	1.75E-02	JAK2
Endometrial Cancer Signaling	2.66E-01	1.67E-02	FOXO3
Primary Immunodeficiency Signaling	2.66E-01	1.56E-02	CD40
mTOR Signaling	2.64E-01	1.41E-02	RHOH,RHOB,RND3
Gαi Signaling	2.63E-01	1.48E-02	CXCR2,ADORA3
IL-2 Signaling	2.6E-01	1.64E-02	FOS
Role of CHK Proteins in Cell Cycle Checkpoint Control	2.5E-01	1.69E-02	CDKN1A
EGF Signaling	2.45E-01	1.56E-02	FOS
Cellular Effects of Sildenafil (Viagra)	2.4E-01	1.29E-02	NOS3, PDE4B
Regulation of Cellular Mechanics by Calpain Protease	2.4E-01	1.37E-02	EZR
Phospholipases	2.35E-01	1.47E-02	PLA2G4A
Superpathway of Melatonin Degradation	2.26E-01	1.23E-02	SMOX
Serotonin Degradation	2.21E-01	1.28E-02	SMOX
Eicosanoid Signaling	2.13E-01	1.16E-02	PLA2G4A

Antiproliferative Role of Somatostatin Receptor 2	2.09E-01	1.39E-02	CDKN1A
Non-Small Cell Lung Cancer Signaling	2.05E-01	1.2E-02	FOXO3
Pyridoxal 5'-phosphate Salvage Pathway	2.01E-01	1.32E-02	SGK1
Mitotic Roles of Polo-Like Kinase	1.97E-01	1.35E-02	PLK2

Part b: Pathways down-regulated

**Ingenuity Canonical Pathways**	**-log (p-value)**	**Ratio**	**Molecules**
PXR/RXR Activation	2.28E00	2.17E-02	ABCC2,CPT1A
FXR/RXR Activation	2.07E00	1.82E-02	ABCC2,MTTP
Mitochondrial L-carnitine Shuttle Pathway	1.57E00	4.55E-02	CPT1A
Granulocyte Adhesion and Diapedesis	1.49E00	1.1E-02	CCL24,PECAM1
Agranulocyte Adhesion and Diapedesis	1.44E00	1.04E-02	CCL24,PECAM1
LPS/IL-1 Mediated Inhibition of RXR Function	1.31E00	8.16E-03	ABCC2,CPT1A
Complement System	1.28E00	2.86E-02	C4BPA
Erythropoietin Signaling	9.87E-01	1.27E-02	CBL
Chemokine Signaling	9.75E-01	1.33E-02	CCL24
Ephrin B Signaling	9.46E-01	1.22E-02	CBL
FLT3 Signaling in Hematopoietic Progenitor Cells	9.41E-01	1.27E-02	CBL
RANK Signaling in Osteoclasts	8.81E-01	1.03E-02	CBL
Fcγ Receptor-mediated Phagocytosis in Macrophages and Monocytes	8.54E-01	9.43E-03	CBL
T Cell Receptor Signaling	8.37E-01	9.17E-03	CBL
Telomerase Signaling	8.2E-01	9.43E-03	TPP1
HIF1α Signaling	8.13E-01	8.93E-03	EGLN1
CCR3 Signaling in Eosinophils	7.69E-01	7.46E-03	CCL24
14-3-3-mediated Signaling	7.62E-01	8.26E-03	CBL
PTEN Signaling	7.58E-01	7.19E-03	CBL
Gαi Signaling	7.45E-01	7.41E-03	NPR3
PI3K Signaling in B Lymphocytes	7.26E-01	6.99E-03	CBL
Insulin Receptor Signaling	7.14E-01	6.71E-03	CBL
AMPK Signaling	7.06E-01	5.52E-03	CPT1A

Hepatic Cholestasis	6.94E-01	5.46E-03	ABCC2
Mitochondrial Dysfunction	6.26E-01	4.65E-03	CPT1A
Acute Phase Response Signaling	6.19E-01	5.52E-03	C4BPA
NRF2-mediated Oxidative Stress Response	5.95E-01	5.13E-03	ABCC2
Clathrin-mediated Endocytosis Signaling	5.85E-01	5.05E-03	CBL
Leukocyte Extravasation Signaling	5.6E-01	4.76E-03	PECAM1
Actin Cytoskeleton Signaling	5.31E-01	4.13E-03	SSH1
Role of Osteoblasts, Osteoclasts and Chondrocytes in Rheumatoid Arthritis	5.21E-01	4E-03	CBL
cAMP-mediated signaling	5.21E-01	4.42E-03	NPR3
Systemic Lupus Erythematosus Signaling	5.18E-01	3.92E-03	CBL
Protein Ubiquitination Pathway	4.68E-01	3.7E-03	CBL
G-Protein Coupled Receptor Signaling	4.64E-01	3.62E-03	NPR3
Xenobiotic Metabolism Signaling	4.47E-01	3.47E-03	ABCC2
Molecular Mechanisms of Cancer	3.68E-01	2.58E-03	CBL
